# Antibody targeting of a specific region of *Pfs47* blocks *Plasmodium falciparum* malaria transmission

**DOI:** 10.1038/s41541-018-0065-5

**Published:** 2018-07-10

**Authors:** Gaspar E. Canepa, Alvaro Molina-Cruz, Lampouguin Yenkoidiok-Douti, Eric Calvo, Adeline E. Williams, Martin Burkhardt, Fangni Peng, David Narum, Martin J. Boulanger, Jesus G. Valenzuela, Carolina Barillas-Mury

**Affiliations:** 1 Laboratory of Malaria and Vector Research, National Insti6tute of Allergy and Infectious Diseases, National Institutes of Health, Rockville, MD 20852 USA; 2 Fischell Department of Bioengineering, University of Maryland, College Park, MD 20742 USA; 3 Laboratory of Malaria Immunology and Vaccinology, National Institute of Allergy and Infectious Diseases, National Institutes of Health, Rockville, MD 20852 USA; 4 Department of Biochemistry and Microbiology, University of Victoria, Victoria, BC V8P 5C2 Canada

## Abstract

Transmission-blocking vaccines are based on eliciting antibody responses in the vertebrate host that disrupt parasite development in the mosquito vector and prevent malaria transmission. The surface protein Pfs47 is present in *Plasmodium falciparum* gametocytes and female gametes. The potential of Pfs47 as a vaccine target was evaluated. Soluble full-length recombinant protein, consisting of three domains, was expressed in *E. coli* as a thioredoxin fusion (T-Pfs47). The protein was immunogenic, and polyclonal and monoclonal antibodies (mAb) were obtained, but they did not confer transmission blocking activity (TBA). All fourteen mAb targeted either domains 1 or 3, but not domain 2 (D2), and immune reactivity to D2 was also very low in polyclonal mouse IgG after T-Pfs47 immunization. Disruption of the predicted disulfide bond in D2, by replacing cysteines for alanines (C230A and C260A), allowed expression of recombinant D2 protein in *E. coli*. A combination of mAbs targeting D2, and deletion proteins from this domain, allowed us to map a central 52 amino acid (aa) region where antibody binding confers strong TBA (78-99%). This 52 aa antigen is immunogenic and well conserved, with only seven haplotypes world-wide that share 96–98% identity. Neither human complement nor the mosquito complement-like system are required for the observed TBA. A dramatic reduction in ookinete numbers and ookinete-specific transcripts was observed, suggesting that the antibodies are interacting with female gametocytes and preventing fertilization.

## Malaria vaccines: Targeting transmission

Transmission blocking activity (TBA) of malaria vaccines can potentially target parasite stages either within the human host or the mosquito vector. A team led by Carolina Barillas-Mury at the National Institutes of Health, USA, engineered a vaccine targeting the protein Pfs47 which is present mainly on the surface of *Plasmodium falciparum* gametocytes. Full-length Pfs47 elicits no TBA; however, an antigen based on a modified version of the central region of Pfs47’s domain 2 generates antibodies with potent TBA in mice. This TBA is independent of human complement or a mosquito complement-like system. Instead, such TBA antibodies likely function by interfering with female gametocytes and preventing fertilization. The Pfs47 region eliciting potent TBA is highly conserved in *P. falciparum*, suggesting the possibility of a broadly-effective vaccine or synergy with other vaccine target antigens.

## Introduction

Malaria, a life-threatening disease caused by *Plasmodium* parasites, is transmitted by anopheline mosquitoes. Although the global malaria mortality rate decreased by 48% between 2000–2015, with an estimated 4.2 million lives saved as a result of scale-up of malaria control interventions, there were still 212 million new cases and an estimated 429,000 malaria-related deaths in 2015.^1^ These gains are threatened as *Plasmodium* parasites around the world exhibit growing resistance to anti-malarial drugs and as mosquitoes become resistant to insecticides.^1^ Effective anti-malarial vaccines are not currently available, and reducing the rate of disease transmission is one of the key steps to control and, eventually, to eradicate malaria.^1^

Mosquitoes become infected when they ingest *Plasmodium* gametocytes as they feed on blood from a malaria-infected host. Both male and female gametes emerge from infected red blood cells in the lumen of the mosquito midgut. Male gametocytes undergo exflagellation and release eight highly motile flagellated microgametes, while female gametocytes mature into macrogametes. Fertilization occurs in the midgut lumen, giving rise to zygotes that mature into motile ookinetes that traverse the mosquito midgut. Those ookinetes that reach the midgut basal lamina transform into oocysts and multiply repeatedly, giving rise to thousands of sporozoites. When the oocyst ruptures, sporozoites are released into the hemocele, migrate to the salivary gland of the mosquito, and are injected into a new vertebrate host when the mosquito ingests the next blood meal.^2^

Sexual stages of *Plasmodium* in the mosquito midgut are vulnerable targets to block malaria transmission, because as parasites emerge from erythrocytes, they become accessible to intervening agents, such as antibodies or human complement, present in the ingested blood. Furthermore, *Plasmodium* development in the mosquito results in population bottlenecks, because parasites suffer great losses in each of the transitions from gametocytes to gametes, zygotes, ookinetes, and finally to oocysts.^3^

Malaria transmission-blocking vaccines rely on functional antibodies present in the serum of the vertebrate host that are ingested by mosquitoes together with *Plasmodium* gametocytes. These antibodies interact with proteins present on the surface of sexual and sporogonic stages of *Plasmodium* or on the surface of the mosquito midgut, and disrupt molecular interactions, such as fertilization, critical for malaria transmission.^3^ Pre-clinical studies led to the development of several *P. falciparum* transmission-blocking vaccine candidates, of which Pfs230, Pfs25, and Pfs48/45 are the best characterized antigens.^4^ Pfs25 protein, expressed on the surface of female gametes in the mosquito midgut, persists throughout the zygote, ookinete, and early oocyst stages.^5^ Pfs230 and Pfs48/45, members of the 6-cysteine family of proteins, are expressed on the surface of both male and female gametocytes in the human host, persist in gametes, and mediate interactions critical for fertilization.^6^

Activation of mosquito immune responses greatly limits *Plasmodium* infection. Ookinete midgut invasion causes irreversible damage to epithelial cells and activates a two-step nitration response regulated by the c-Jun N-terminal Kinases (JNK) pathway. This epithelial nitration triggers the local release of hemocyte-derived microvesicles which promotes mosquito complement activation.^7^ The thioester-containing protein 1 (TEP1), a key mediator of the mosquito complement-like system, binds to the ookinete surface and forms a complex that ultimately kills the parasite. Using linkage mapping and functional genetics, we identified *Pfs47* as a *P. falciparum* gene that allows parasites to evade the mosquito immune system.^8^ Pfs47 is also a member of the 6-cysteine family of proteins and is expressed on the surface of female gametocytes, gametes, zygotes, and ookinetes.^9^

Parasites expressing a Pfs47 haplotype compatible with a given mosquito vector disrupt JNK signaling in the invaded midgut cells, which prevents an effective nitration response and allows the ookinete to evade TEP1-mediated killing.^10^ The fact that *P. falciparum* has evolved the capacity to evade mosquito immunity suggests that these responses are an important barrier to malaria transmission. Although there are clear Pfs47 orthologs in other *Plasmodium* species, the sequence homology is low. P47 is critical for mosquito immune evasion and for optimal fertilization in *P. berghei* (murine malaria), but there is no clear evidence of involvement in *P. falciparum* fertilization.^9^

Previous studies showed that three monoclonal antibodies (mAbs) against Pfs47 had no effect on *P. falciparum* infection in *A. stephensi* mosquitoes.^11^ However, studies with two other transmission-blocking vaccine targets (Pfs230 and *A. gambiae* aminopeptidase N) found that the region of the protein where antibodies bind is critical to block transmission.^12,13^ Polyclonal antibodies obtained after DNA vaccination of mice with a *P. vivax* P47 (Pvs47) coding plasmid significantly reduced *Plasmodium vivax* infection of *Anopheles dirus.*^14^ In this work, we explored the potential of Pfs47 as vaccine target, and used a combination of polyclonal and mAbs and protein deletions to search for targets to block *P. falciparum* infection in *A. gambiae* mosquitoes. A 52 amino acid (aa) region of Pfs47 where antibody binding confers strong (78–99%) TBA was identified, and the mechanism by which this interaction prevents mosquito infection was explored.

## Results

### Antibodies to full-length Pfs47 (T-Pfs47) do not confer TBA

**Fig. 1:**
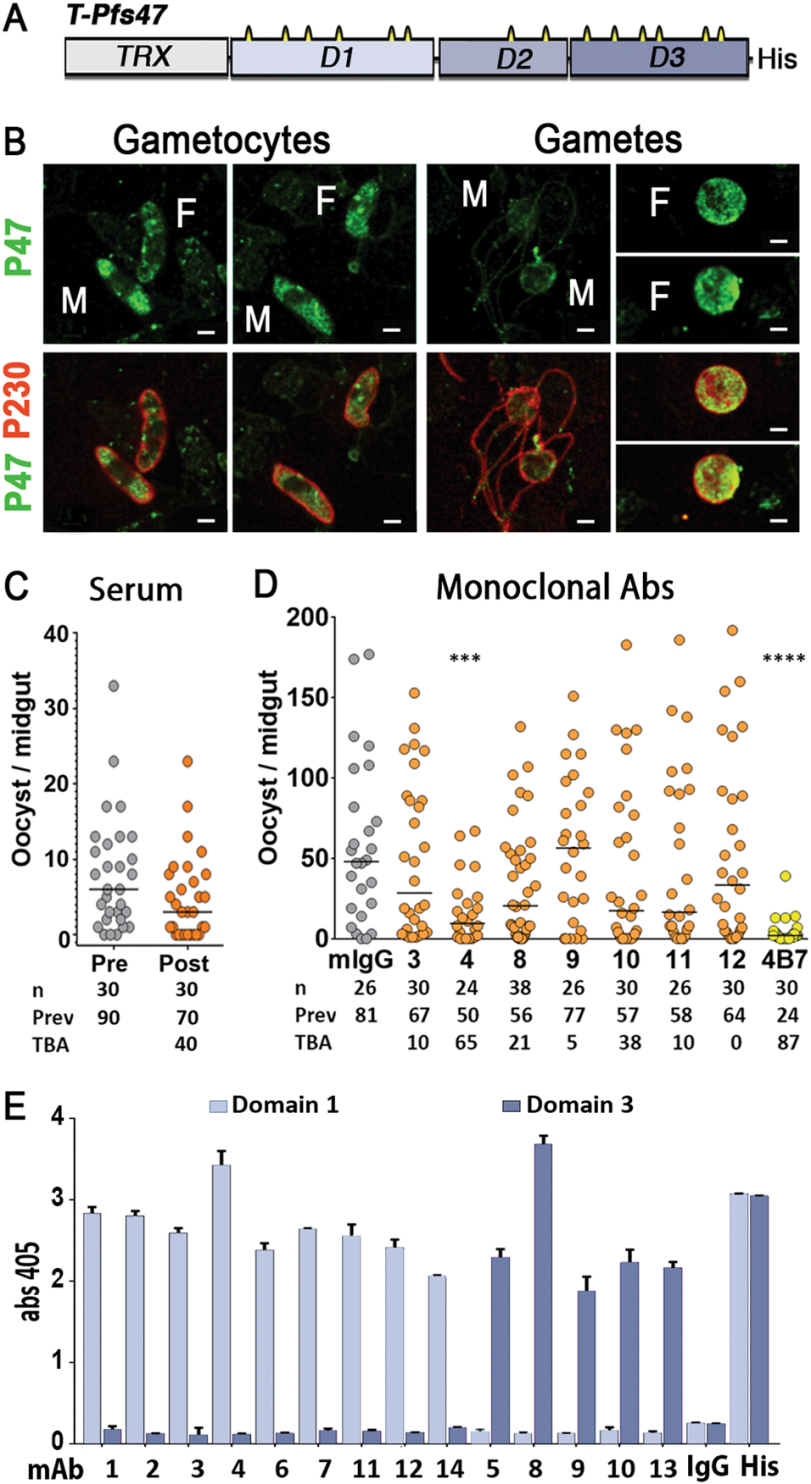
Expression, immunogenicity and transmission blocking activity of polyclonal and monoclonal antibodies against T-Pfs47 recombinant protein. **a** Schematic representation of *P. falciparum* T-Pfs47 fusion protein. The D1 and D3 domains have 6 cysteines (in yellow), while the D2 domain only has 2 cysteines, “His*”* denotes six-histidine tag. The complete protein and optimized DNA sequence of T-Pfs47 is included in Fig. S2. **b** In vitro cultured *P. falciparum* NF54 gametocytes and gametes were stained with purified total IgG against T-Pfs47 (P47 in green) or antisera against Pfs230 (P230 in red). F, female, M, male. Scale bars, 2 μm. **c** Malaria transmission blocking activity (TBA) assay. Total IgG was purified from serum collected from mouse 2 at day 50 post-immunization. IgG (200 μg/ml) was mixed with *P. falciparum* NF54 cultured gametocytes and fed to *A. gambiae* mosquitoes in standard membrane feeding assays (SMFA). **d** TBA of IgG (200 μg/ml) from monoclonal antibodies (mAbs) against T-Pfs47. In **c** and **d** the dots represent the number of oocysts in individual mosquitoes and the lines indicate the medians. Number of mosquitoes dissected (*n*); Infection prevalence (Prev); TBA was calculated as percent inhibition of infection intensity in an SMFA relative to IgG from naive mice (mIgG); mAb 4B7 against Pfs25 was used as positive control. Medians were compared using the Mann–Whitney test: asterisks indicate statistically significant differences; *P < 0.05; **P < 0.01; ***P < 0.001; ****P < 0.0001. **e** Immunoreactivity of mAbs to T-Pfs47 (1 to 14 at 0.1 μg/ml) against recombinant T-Pfs47-D1 (Domain 1) or T-Pfs47-D3 (Domain 3) (1 μg/ml) proteins. The complete protein and optimized DNA sequences of T-Pfs47-D1 and T-Pfs47-D3 are included in Fig. S7. Column and errors bars represent mean OD 405 ± standard deviation of three replicate ELISA assays, "His" indicates detection with a mAb antibody against six-histidine tag

*P. falciparum* Pfs47 is a surface protein with three s48/45 domains.^9^ Two of them (D1 and D3) each have six cysteines predicted to confer the canonical s48/45 structural fold of this protein family; while domain 2 (D2) is a degenerate domain with only two cysteine residues (Fig. 1a, top). The high number of cysteine residues in Pfs47 (a total of 14) makes it very difficult to obtain a properly folded and stable recombinant protein in which all of the disulfide bonds are properly formed. We attempted to produce soluble recombinant Pfs47 protein using different heterologous systems, but expression of several different Pfs47 constructs in mammalian (Human Embryonic Kidney cells) or insect cell lines (*Anopheles gambiae* Sua5.1 and *Aedes aegypti* Aag2 cells) was unsuccessful (Table S1).

We were able to produce stable and soluble Pfs47 protein using the Baculovirus expression system (BV-Pfs47), but the protein yield was very low (about 100 µg of recombinant protein/L of harvested supernatant) (Fig. S1 A, B). Finally, expression of full-length Pfs47 as a fusion protein with thioredoxin (T-Pfs47) (Fig. 1a, Fig. S2 A, B) in *Escherichia coli* resulted in high protein yield (about 1 mg/L) in inclusion bodies. An in-column HPLC nickel affinity purification/refolding protocol was developed and pure soluble fusion protein was obtained (Fig. S1).

BALB/c mice were immunized with purified T-Pfs47 protein to assess the immunogenicity of this potential antigen. Antibody responses were monitored by performing ELISA with mouse serum obtained at regular intervals during the immunization protocol. All immunized mice developed high antibody titers (Fig. S3). Serum from immunized mice stained the cytoplasm of both male and female *P. falciparum* gametocytes (Fig. 1b). The staining became more prominent in activated female gametes and was present on both the surface and the cytoplasm. In contrast, only weak staining was detected in the remnants of exflagellating male gametes, and Pfs47 could not be detected in mature microgametes (Fig. 1b). Serum from two immunized mice (mouse 2, Fig. 1c and mouse 3 Fig.S3) had modest transmission blocking activity (TBA) (40% and 32%, respectively) in standard membrane feeding assays (SMFA) in *A. gambiae* (Fig. 1c and Fig S3); while serum from mice 1 and 4 were either negative or had minimal (12%) TBA, respectively (Fig. S4). To further assess the potential of T-Pfs47 as a transmission blocking antigen, mice 2 and 3 received an additional immunization and were used in two independent hybridoma fusions to obtain anti-T-Pfs47 mAbs. Fourteen independent mAbs positive in ELISA against BV-Pfs47, a soluble protein that lacks the thioredoxin fusion (Fig. S5) were obtained, but only five of them stained gametocytes (Fig. S6), indicating that they recognize epitopes of Pfs47 accessible in the protein conformation present in the parasite. All mAbs were tested for TBA, but only four mAbs (mAbs 1, 2, 4, and 5) exhibited modest TBA, that was variable (Fig. 1d, Table S2). The rest of the mAbs did not have any detectable TBA (Fig. 1d, Table S2). When four mAbs with the highest ELISA titers (mAbs 2, 3, 4, and 8) were combined in equal ratios, significant TBA (60 and 71%) was observed in two independent assays (Table S3); however, in two additional experiments, there was no significant effect (Table S3).

### Antibodies to full-length Pfs47 (T-Pfs47) target Domains 1 and 3, but not Domain 2

To establish whether the three Pfs47 domains were eliciting antibody responses when mice were immunized with the full-length T-Pfs47 protein, each individual domain was expressed. Thioredoxin fusion proteins for Domains 1 and 3 (T-Pfs47-D1 and T-Pfs47-D3) were readily obtained in *E. coli* (Fig. S7). The Domain 2 (D2) fusion protein, however, could not be expressed after multiple attempts, as bacteria expressing the T-Pfs47-D2 protein construct failed to grow, even before expression was induced with IPTG, suggesting that D2 is toxic to *E. coli*. Nine mAbs recognized D1 and the other five D3 (Fig. 1e), indicating that none of the fourteen mAbs recognized D2. All antibodies also recognized BV-Pfs47 and a second recombinant protein, a D1-D3 fusion in which D2 was removed (Fig. S8, Fig. S9).

**Fig. 2:**
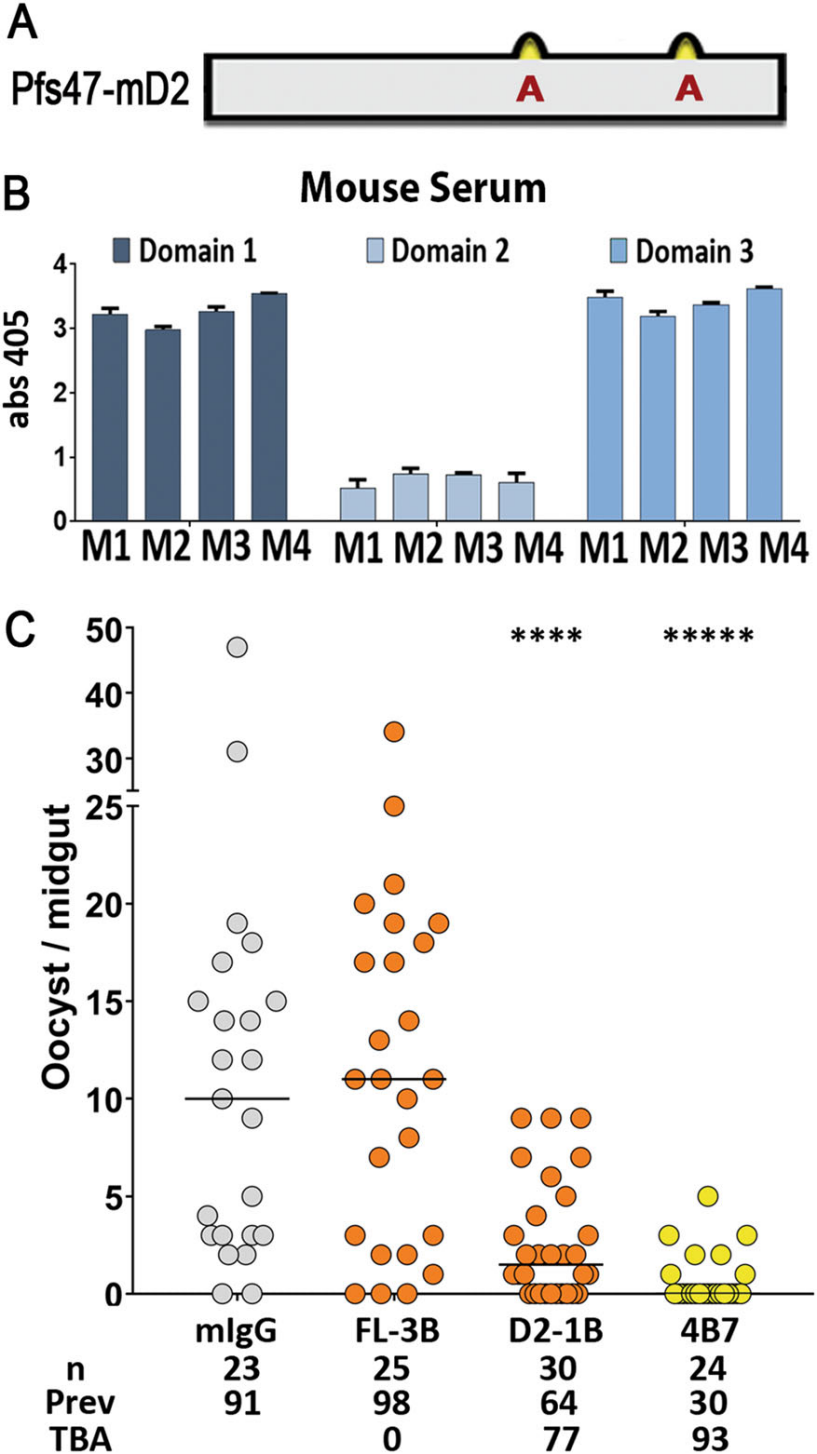
Expression and immunoreactivity of T-Pfs47 polyclonal antibodies against individual domains of Pfs47 and transmission blocking activity of Pfs47-mD2 antibodies in *A. gambiae*. **a** Schematic representation of *Plasmodium falciparum* Pfs47-mD2 recombinant protein. The D2 domain has 2 cysteines (in yellow), amino acid substitutions to alanines (C230A and C260A) in Pfs47-mD2 are shown in red. The complete protein and optimized DNA sequence of T-Pfs47-mD2 is included in Fig. S10. **b** ELISA reactivity of sera from four mice immunized with T-Pfs47 against Pfs47 domains 1, 2, and 3 (T-Pfs47-D1, Pfs47-mD2, T-Pfs47-D3, respectively), column and errors bars represent mean OD 405 ± standard deviation of three replicate assays. **c** TBA using 200 μg/ml of IgG from mice immunized with T-Pfs47 after three boosts (FL-3B) or with Pfs47-mD2 after one boost (D2-1B) in SMFA. Dots represent the number of oocysts in individual mosquitoes and the lines indicate the median. Number of mosquitoes dissected (*n*); Infection prevalence (Prev); TBA was calculated as percent inhibition of infection intensity in an SMFA relative to IgG control purified from naïve mice (mIgG); mAb 4B7 against Pfs25 was used as positive control. Medians were compared using the Mann–Whitney test, asterisks indicate statistically significant differences; ****P < 0.0001

The hypothesis that the lack of transmission blocking was due to the inability of full-length Pfs47 protein to elicit antibody responses to D2 was investigated. D2 has two cysteines predicted to form a disulfide bond that would stabilize a loop structure. The effect of disrupting this loop on the toxicity to *E. coli* was investigated by replacing the cysteine residues with alanines (C230A and C260A, Fig. 2a, Fig. S10). These amino acid substitutions eliminated toxicity and allowed high levels of modified Pfs47-D2 (Pfs47-mD2) expression in *E. coli* (Fig. S10). ELISA assays with sera from four mice immunized with T-Pfs47 confirmed that the D1 and D3 domains elicit much stronger antibody responses than mD2 (Fig. 2b). Hence, vaccination of mice with T-Pfs47 leads to antibodies that target domains D1 and D3, but not D2, and have poor TBA.

### Anti-Pfs47 antibodies that target a specific region of Domain 2 block malaria transmission

Purified Pfs47-mD2 fusion protein (Ser155-Gln267, C230A, and C260A) was used to immunize mice. Purified mouse IgG obtained after one immunization boost with Pfs47-mD2 significantly reduced the median number of oocysts present, relative to the IgG control in *A. gambiae* (Mann–Whitney, *p* < 0.0001), reducing transmission by 77%, while antibodies after immunization with T-Pfs47 had no significant effect, even after three immunization boosts (Fig. 2c).

**Fig. 3:**
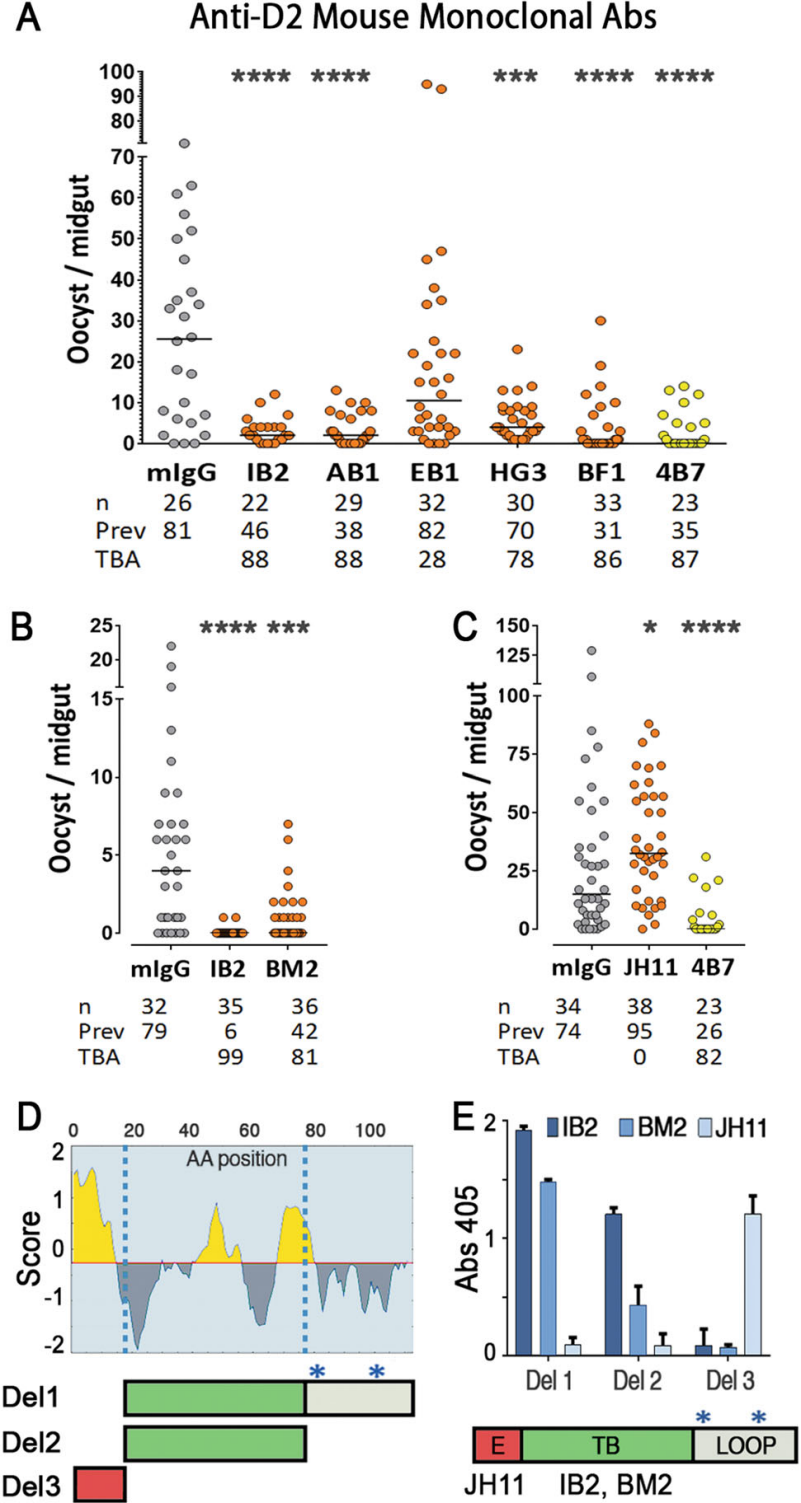
Transmission blocking activity (TBA) and epitope analysis of mAbs obtained after Pfs47-mD2 immunization. **a** TBA of IgG (200 μg/ml) from mAbs against Pfs47-mD2. **b**, **c** TBA of mAbs (200 μg/ml) against Pfs47-mD2 (IB2, BM2, and JH11) from stable hybridoma cultures. Dots represent the number of oocysts in individual mosquitoes and the lines indicate the median. Number of mosquitoes dissected (*n*); Infection prevalence (Prev); TBA was calculated as percent inhibition of infection intensity in an SMFA relative to IgG from naïve mice (mIgG); mAb 4B7 against Pfs25 was used as positive control. Medians were compared using the Mann–Whitney test: asterisks indicate statistically significant differences *P < 0.05; **P < 0.01; ***P < 0.001; ****P < 0.0001. **d** Top, Linear B-cell epitopes prediction in Pfs47-mD2 based on Bepipred; Bottom, schematic representation of Pfs47-mD2 deletion constructs (Del 1, Del 2, Del 3) used to determine the regions were mAbs bind to Pfs47-mD2, blue asterisks indicate cysteines substituted with alanines. The complete protein and optimized DNA sequences of Pfs47-mD2 deletion constructs (Del 1, Del 2, Del 3) are included in Fig. S11. **e** Top, immunoreactivity of mAbs JH11, IB2, and BM2 (0.1 μg/ml) to Pfs47-mD2 deletions 1 to 3 (1 μg/ml). Column and errors bars represent mean OD 405 ± standard deviation of three replicate assays. Bottom, schematic representation of regions of Pfs47-mD2 where antibody binding enhanced infection (E), or blocked transmission (TB); C-terminal region containing the cysteine loop of Domain 2 (LOOP)

Mice immunized with Pfs47-mD2 were also used for two independent hybridoma fusions, and eight independent mAbs that recognize soluble Pfs47-mD2 were obtained. Based on ELISA and Western blot analysis, some mAbs recognize linear epitopes, while others target conformational ones (Table S4). Interestingly, only four mAbs that bind to Pfs47-mD2 by ELISA also recognize BV-Pfs47 protein, indicating that some mD2 epitopes are not exposed in the full-length protein and are probably masked by D1 or D3. Purified IgG from each mAb (200 µg/ml) was tested at least twice for TBA in four independent experiments (Fig. 3a shows Experiment #2, all experiments are shown in Table S4). Four independent mAbs (IB2, HG3, BF1, BM2) had strong reproducible TBA (Fig. 3a and Table S4), while the TBA of AB1 was modest (37%) in a replicate experiment (Table S4). Of the eight hybridomas, only lines IB2, BM2, and JH11 were stable. Strong TBA was confirmed for IB2 (99%) and BM2 (81%) in an additional experiment (Fig. 3b). In contrast, JH11 mAb significantly enhanced infection, doubling the median number of oocysts in two independent experiments relative to control IgG (Fig. 3c, Table S4).

IB2, BM2, and JH11 recognize BV-Pfs47 protein under denaturing conditions (Table S4), indicating that they bind to linear epitopes. Linear B cell epitopes in Pfs47-mD2 were predicted using the Bepipred Linear Epitope Prediction^15^ (Fig. 3d) to generate three protein deletions (Fig. 3d, S9) and to determine the regions of mD2 where antibody binding blocks transmission (IB2 and BM2) or enhances (JH11) *Plasmodium* infection. ELISA assays with the three mD2 deletion proteins (Del 1–3) revealed that JH11 binds the N-terminal epitope region (E = enhanced infection region, red), while IB2 and BM2 bind in the central regions (TB = transmission blocking region, green) upstream of the modified cysteine loop (Fig. 3e).

**Fig. 4:**
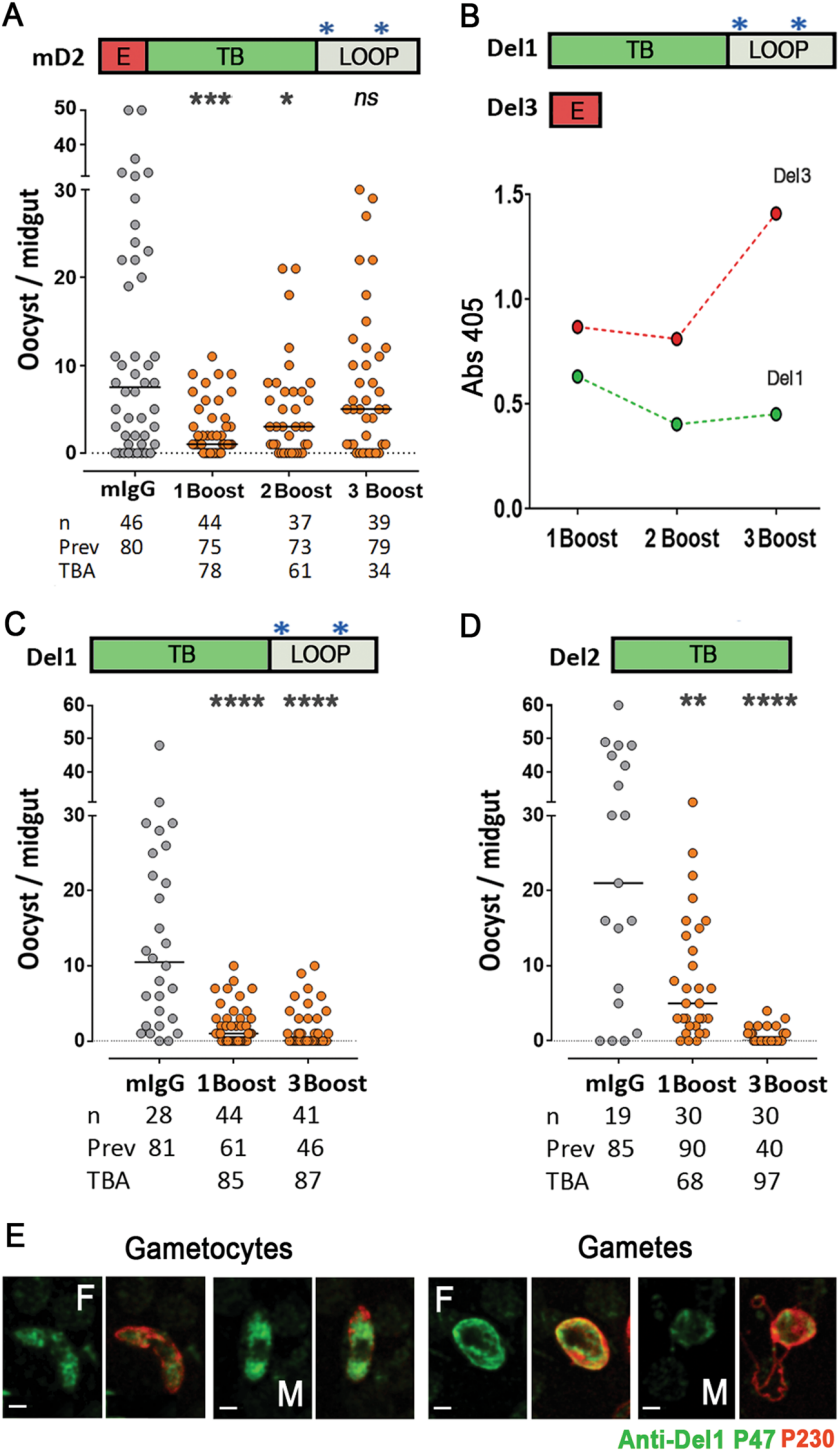
Immunoreactivity and transmission blocking activity (TBA) of polyclonal sera against modified Pfs47-D2 (Pfs47-mD2), Pfs47-mD2-Del1, and Pfs47-mD2-Del2. **a** Top, Schematic representation of Pfs47-mD2, region that enhanced infection (E), transmission blocking region (TB) and cysteine Loop (Loop); Bottom, TBA of mouse polyclonal IgG (200 μg/ml) against Pfs47-mD2 after 1, 2 or 3 immunization boosts. **b** Top, Schematic representation of Pfs47-mD2 Del1 and Del3; Bottom, immunoreactivity of polyclonal sera after 1, 2 or 3 immunization boosts with Pfs47-mD2 to Pfs47-mD2-Del1 (Del1) and Pfs47-mD2-Del2 (Del2) peptides by ELISA. Dots and errors bars represent mean OD 405 ± standard deviation of three replicate assays. **c** Transmission blocking activity of polyclonal sera against Pfs47-mD2 Del1 (200 μg/ml) after 1 or 3 boosts. **d** Transmission blocking activity of polyclonal sera against Pfs47-mD2 Del2 (200 μg/ml) after 1 or 3 boosts. **e** In vitro cultured *P. falciparum* NF54 gametocytes and gametes were stained with total IgG against Pfs47-mD2 Del1 (P47 in green) or antisera against Pfs230 (P230 in red). F female, M male. Scale bars, 2 μm. Dots in **a**, **c** and **d** represent the number of oocysts in individual mosquitoes and the line indicates the median. Number of mosquitoes dissected (*n*); Infection prevalence (Prev); Transmission blocking activity (TBA) as percent inhibition of infection intensity in an SMFA relative to IgG from naive mice (mIgG); mAb 4B7 against Pfs25 was used as positive control. Medians were compared using the Mann–Whitney test: asterisks indicate statistically significant differences; *P < 0.05; ****P < 0.0001

The immunogenicity of Pfs47-mD2 was further characterized. Surprisingly, the TBA of purified IgG decreased as mice received additional immunizations with Pfs47-mD2 (Fig. 4a). ELISA assays revealed that the N-terminal region that enhances infection (“E”, red) is immunodominant, and this effect becomes stronger with subsequent immunizations (Fig. 4b). Removal of this immunodominant N-terminal region from the antigen (Del 1) preserved TBA throughout the immunization scheme (Fig. 4c) and resulted in strong TBA (87–99%). Immunization with the 52 aa TB region (Del 2) gave similar results with TBA of 78–97% (Fig. 4d, Fig. S10). Furthermore, antibodies against the Del1 antigen also stain the cytoplasm of both male and female gametocytes (Fig. 4e); and we confirmed that the Pfs47 signal is more prominent in activated female gametes but cannot be detected in mature microgametes (Fig. 1b).

Pfs47 is a polymorphic gene, and a previous analysis of 364 global *P. falciparum* isolates identified 42 different protein haplotypes, with the D2 being the most polymorphic region.^10^ However, most polymorphisms are present in the cysteine loop region of D2, while the central region, where antibody binding has strong TBA (Fig. 4c, d, TB region in green), is highly conserved. Reanalysis of this 52 aa region from the global Pfs47 sequences identified only seven different Pfs47 haplotypes, six of which differ by a single amino acid (98% identity) and one of which differs by two amino acids (96% identity) (Fig. S13).

**Fig. 5:**
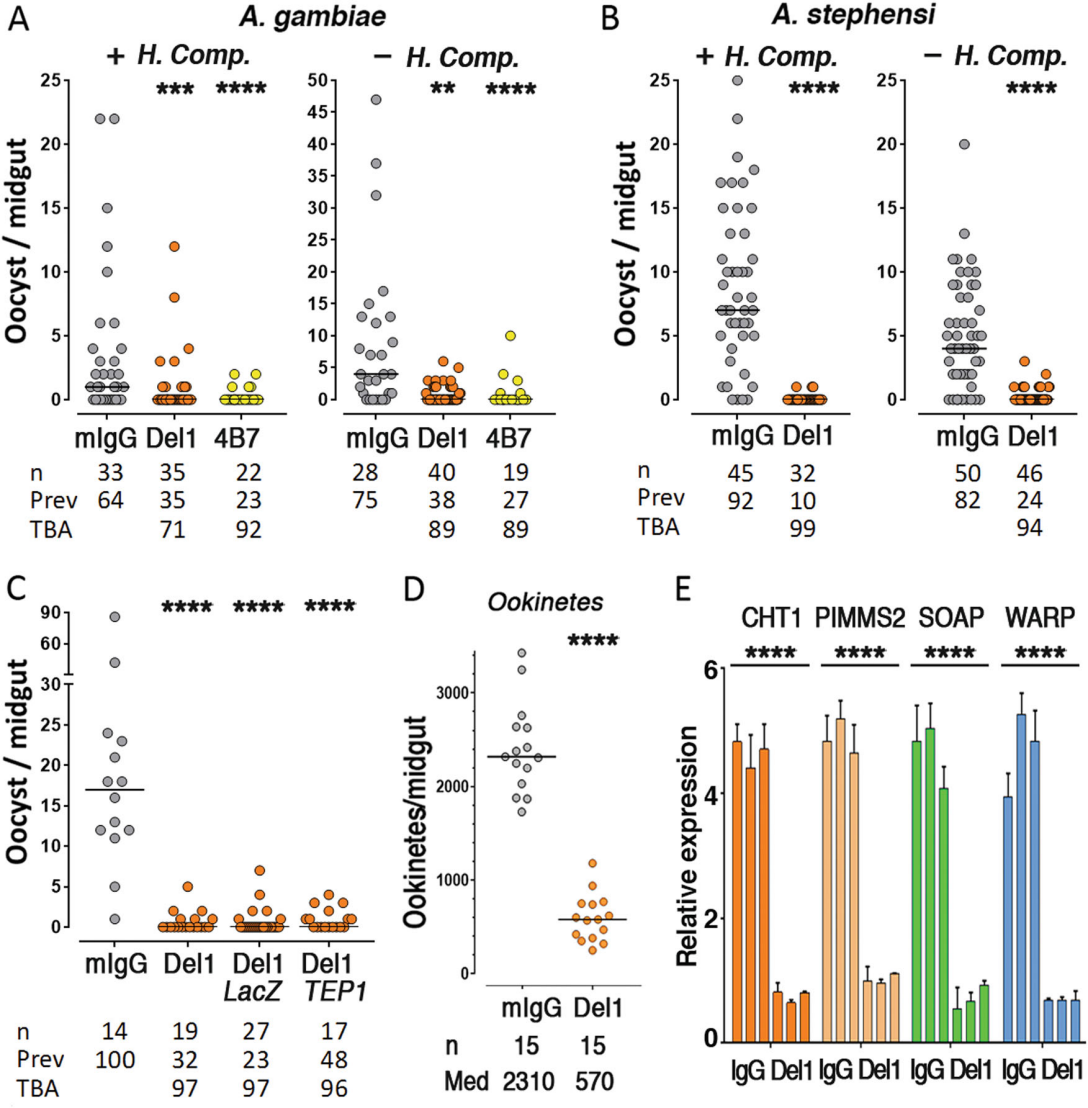
Effect of human complement and mosquito complement-like system on transmission blocking activity (TBA), and mechanism of action of antibodies against Pfs47-mD2 Del1. TBA of polyclonal sera against Pfs47-mD2 Del1 (300 μg/ml) in the presence (+) or absence (−) of Human Complement (H.Comp) in *A. gambiae* (**a**) or *A. stephensi* (**b**). **c** Effect of silencing of TEP1 in *A. gambiae* on transmission blocking activity of polyclonal sera against Pfs47-mD2 Del1 (300 μg/ml), dsLacZ was injected as a negative control. Dots in (**a**–**c**) represent the number of oocysts in individual mosquitoes and the line indicates the median. Number of mosquitoes dissected (*n*); Infection prevalence (Prev); TBA was calculated as percent inhibition of infection intensity in an SMFA relative to IgG from naïve mice (mIgG); mAb 4B7 against Pfs25 was used as positive control. Medians were compared using the Mann–Whitney test: asterisks indicate statistically significant differences, **P < 0.01; ***P < 0.001; ****P < 0.0001. Effect of polyclonal sera against Pfs47-mD2 Del1 (300 μg/ml) on ookinete formation (**d**) and (**e**) expression of ookinete-specific genes in *A. gambiae* 24 h post infection. Data points represent the number of ookinetes in individual mosquitoes and the lines indicate the medians. Number of mosquitoes dissected (*n*); Medians (Med) were compared using the Mann–Whitney test: ****P < 0.0001. CHT1 chitinase 1, PIMMS2 *Plasmodium* invasion of mosquito midgut screen candidate 2, SOAP secreted ookinete adhesive protein, WARP von Willebrand factor A domain-related protein. *P* < 0.0001, mean levels of expression were compared using ANOVA

We investigated whether human complement or the mosquito complement-like system mediated the TBA observed with anti-Pfs47 mD2-Del1 polyclonal mouse IgG. There was no significant difference in TBA in the presence (+H. Comp.) or absence (-H. Comp) of human complement in both *A. gambiae* and *A. stephensi* mosquitoes (Fig. 5a, b). Disruption of the *A. gambiae* immune system by silencing the TEP1, a key effector of the complement-like system, also had no effect on the level of TBA observed (Fig. 5c). In *P. berghei*, *P47* is required for female fertility under in vitro culture conditions, and disruption of the gene also significantly impairs fertilization in vivo*.*^16^ Moreover, *Pfs47* is localized on the surface of female gametocytes and gametes, as well as of zygotes and ookinetes.^9^ We investigated whether anti-Pfs47 mD2-Del1 antibodies disrupt *Plasmodium* fertilization and thus reduce ookinete formation in *A. gambiae*. Indeed, there was dramatic (75.3%) reduction in the number of ookinetes present in the midgut lumen 24 h post-feeding (*P* < 0.0001, Mann–Whitney) (Fig. 5d), as well as in the expression level of four ookinete-specific genes^17–20^ (*P* < 0.0001, ANOVA) (Fig. 5e).

## Discussion

Several transmission-blocking *Plasmodium* antigens have been identified over the past decades, including the pre-fertilization proteins Pfs48/45 and Pfs230, members of the six-cysteine family present on the surface of gametes, and the post-fertilization antigen Pfs25.^21^ Previous studies have shown that protective epitopes in these antigens are conformation-dependent.^22^ Producing large amounts of recombinant proteins with the proper folding has been a major challenge, especially for members of the six-cysteine family, because of the multiple disulfide bonds that must be properly formed.^21^ For Pfs47, the use of a thioredoxin carrier and expression in the *E.coli* Shuffle® T7 facilitated disulfide bond formation. Although the recombinant full-length protein accumulated in inclusion bodies, an in-column refolding protocol made it possible to recover pure soluble and stable protein for immunization. Furthermore, the 14 mAbs that were obtained after immunization with the refolded protein expressed in *E. coli* also recognized the recombinant BV-Pfs47, indicating that these two recombinant proteins shared several immunogenic epitopes.

We were disappointed that none of the 14 mAb, nor the purified mouse polyclonal IgG after immunization with the full-length protein, had significant TBA. This is consistent with previous reports that immunization with full-length Pfs47 does not confer TBA.^21^ Although the D2 domain comprises roughly one third of the Pfs47 protein, none of the mAbs raised against full length T-Pfs47 targeted this domain, and even the reactivity of polyclonal mouse IgG to the D2 domain was much lower than to domains D1 or D3. However, the mD2 domain without cysteines is immunogenic, suggesting that the lack of antibody response to this region after immunization with the full-length protein may be due to immunodominant epitopes present in the D1 and D3 domains, and/or to the lack of exposure of the D2 domain due to masking by the other two domains.

mAbs that bind to the central region of mD2, immediately before the cysteine loop region, have strong TBA, while a mAb that binds to the N-terminal region enhances *Plasmodium* infection. Although the precise domain boundaries of Pfs47 are not known, the N-terminal of D2 could serve as a linker between domains 1 and 2. Antibody binding to this region may stabilize a conformation that facilitates the interaction of Pfs47 with a partner in the parasite that is involved in fertilization, or with a mosquito receptor that mediates immune evasion. Interestingly, the N-terminal epitope in mD2 is immunodominant and took over the IgG response after three immunization boosts, resulting in polyclonal mouse IgG with no significant TBA. A new recombinant protein in which this deleterious N-terminal region was removed (mD2-Del1) solved this problem and generated mouse IgG with strong TBA. Furthermore, immunization with a recombinant protein that includes only the central 52 aa region before the cysteine loop region elicited strong TBA. All recombinant proteins in this study were made based on the GB4 Pfs47 haplotype (Hp1, Fig. S13), the most frequent one in West Africa, and all the TBA were done with NF54 parasites that express Hp2 (Fig. S13) that differs from Hp1 by a single amino acid, and cross-protection was observed. The low variability in this 52 aa region, with only seven different haplotypes present world-wide that share 96–98% aa identity, makes it very likely that antibodies to one haplotype will cross-react with others. The cross-reactivity and cross-protection of anti-Pfs47-Hp1 antibodies with other haplotypes is currently under investigation. Due to its small size (52aa), conjugation of this antigen with a protein carrier is likely to enhance immunogenicity.

All immunizations in these studies were done using the Magic Mouse® adjuvant that contains immune-stimulatory short CpG DNA-oligodeoxynucleotides with unmethylated cytosine-guanine dinucleotides. Optimization of adjuvants, expression systems, and delivery strategies would be necessary to move this vaccine forward into clinical trials. The initial hypothesis that antibodies to *Pfs47* would hamper the ability of *P. falciparum* to evade mosquito immunity could not be tested, because parasite development was blocked at a very early stage. Most likely, anti-Pfs47 antibody binding to the surface of female gametes disrupted fertilization. The proposed anti-Pfs47 vaccine appears to block *P. falciparum* infection through a mechanism different from other leading targets, because Pfs47 is expressed on the surface of female gametes, while the anti-Pfs48/45 vaccine targets male gametes; and unlike Pfs230 (which also targets gametes), the Pfs47 TBA is independent of human complement. Moreover, Pfs25 protein is expressed in zygotes and ookinetes, while the observed TBA in Pfs47 appears to be acting at an earlier stage by preventing fertilization. This opens the possibility that the Pfs47 antigen may synergize with other transmission blocking vaccine targets. This 52 aa Pfs47 antigen is a promising vaccine candidate that lacks cysteine residues. Elicitation of protective antibodies does not seem to be conformation-dependent, it can be expressed at high yields in *E.coli*, and is immunogenic and protective.

## Methods

### Recombinant protein

The mature full-length *Plasmodium falciparum* Pfs47 coding sequence extending from the predicted signal peptide cleavage site to the GPI anchor site (Leu28 to Ala414; GenBank: ALQ44015.1) was codon optimized for insect cell expression and synthesized by GenScript (Piscataway NJ). Two forms of Pfs47 were cloned from the mature full length Pfs47. The BV-Pfs47 construct was built by PCR amplification of the Pfs47 coding sequence from Thr32 to Tyr420, that was sub-cloned into a modified pAcSecG2T vector (Pharmingen), containing a TEV protease cleavable N-terminal hexahistidine/maltose binding protein (MBP) tag. The Pfs47 Domain 1 and Domain 3 fusion protein (Pfs47-D1-D3) construct, which lacks a predicted Domain 2 (D2), was cloned by overlap extension PCR to replace the predicted D2 (Ser155 to Gln267) with a GSGGSG tether linker. This Pfs47 D1-D3 construct (Thr32-Asn154 GSGGSG Asn268-Ala414) was subcloned into a modified pAcGP67b vector (BD Biosciences) with a hexahistidine tag preceded by a TEV cleavage site at the C terminus of the protein. Recombinant Pfs47 constructs were produced in insect cells using established protocols.^23^ In brief, high titer expression viruses were generated in *Spodoptera frugiperda* 9 (Sf9) cells and used to infect *Trichoplusia ni* (Hi5) cells. Secreted proteins were harvested from the Hi5 cell culture supernatant after 65 hs of infection and purified by nickel affinity chromatography. Using TEV protease, the hexahistidine/MBP tag was cleaved; and the tag was removed by ion exchange chromatography. Proteins were purified to homogeneity by size exclusion chromatography in HEPES-buffered saline (20 mM HEPES pH 7.5, 150 mM NaCl) containing 2% glycerol.

Alternatively, the Thioredoxin-Pfs47 (T-Pfs47) construct (Thr32 to Tyr420) was codon optimized for *Escherichia coli* expression (provided by Dr. George Christophides), cloned by PCR and subcloned into pET32 plasmid, containing a Thioredoxin N-terminal fusion and a C-terminal hexahistidine tag. T-Pfs47 was expressed in SHuffle® T7 Competent *Escherichia coli*. 8 M urea solution containing inclusion bodies of cultures induced for 3 h at 37 °C with 1 mM isopropyl β-D-thiogalactopyranoside (Sigma) were purified by an in-column HPLC nickel affinity purification/refolding chromatography (Thermofischer) and dialyzed against PBS. Similarly, Pfs47 Domain 1 (Thr32 to Asn 154) and Pfs47 Domain 3 (Asn268 to Ala414) were produced using the same method mentioned above to obtain Thioredoxin-Pfs47 Domain 1 (T-Pfs47-D1) and Thioredoxin-Pfs47 Domain 3 (T-Pfs47-D3).

Expression of recombinant Pfs47 Domain 2 (Ser155 to Gln267) protein alone, or as a thioredoxin fusion in *E. coli* was attempted multiple times, but no bacterial colonies could be obtained after transformation, suggesting that the protein product was toxic to *E. coli*. To overcome this problem, Pfs47 Domain 2 (Ser155 to Gln267) was synthetized in which the two cysteines were replaced by alanines (C230A and C260A). A hexahistidine tag was added at the C-terminal and subcloned into pET17b to obtain Pfs47 modified Domain 2 (Pfs47-mD2). The latter construct was used as template to generate several Pfs47-mD2 deletions: Pfs47-mD2-Del1 (Ile178 to Gln267), Pfs47-mD2-Del2 (Ile178 to Asn229) and Pfs47-mD2-Del3 (Ser155 to Asn181) by PCR amplification of different regions that were subcloned into the pET17b vector. All constructs derived from Pfs47-mD2 were isolated from inclusion bodies, purified by nickel affinity chromatography and dialyzed in HEPES-buffered saline (20 mM HEPES pH 7.5, 150 mM NaCl). All gel images derive from the same experiment and were processed in parallel.

### Mosquitoes and Parasites

*Anopheles gambiae* G3 and *Anopheles stephensi* Nijmegen strains were used. Mosquitoes were reared at 27 °C and 80% humidity on a 12-h light-dark cycle under standard laboratory conditions. The *Plasmodium falciparum* strains NF54 was maintained in O + human erythrocytes using RPMI 1640 medium supplemented with 25 mM HEPES, 50 mg/l hypoxanthine, 25 mM NaHCO_3_, and 10% (v/v) heat-inactivated type O + human serum (Interstate Blood Bank, Inc., Memphis, TN) at 37 °C and with a gas mixture of 5% O_2_, 5% CO_2_, and balance N_2_.^10^

### Mosquito feeding

Mosquito females were infected artificially by membrane feeding with *P. falciparum* gametocyte cultures. Gametocytogenesis was induced as previously described.^10^ Briefly, the RBCs of a 14–17-day old mature gametocyte cultures (stages IV and V) of the *P. falciparum* NF54 line were separated by centrifugation (5 min, 2500 g). The infected RBCs were resuspended in one volume of human serum at 37 °C and then diluted to 0.15%-0.2% stage V gametocytemia with O + human RBC at 42% in human serum at 37 °C. All manipulations were done maintaining the tubes in water at 37 °C. A 244 ul sample of diluted gametocytes was mixed into an Eppendorf tube containing 16 μl of Ab test sample at 37 °C and delivered to a prewarmed (37 °C) glass feeder with parafilm as membrane. The test sample contained protein G purified IgG monoclonal antibodies (mAb), protein G purified mouse polyclonal antibodies, or protein G purified IgGs from control mouse diluted in non- heat inactivated AB + human sera at the mentioned concentrations. Mosquitoes were fed using membrane feeders with a water jacket at 37 °C for 30 min. Midguts were dissected 8–10 d after feeding, and oocysts were stained with 0.05% (wt/vol) mercurochrome in water and counted by light microscopy. The distribution of parasite numbers in individual mosquitoes between control and experimental groups was compared using the nonparametric Mann–Whitney test (GraphPad, Prism). The percent reduction in transmission (TBA) was calculated as follows: 100 × {1−[(mean number of oocysts in the test)/(mean number of oocysts in the control)]}. All standard membrane feeding assays were confirmed in two to three independent experiments. Multiple naive mouse sera was purchased from Sigma-Aldrich (St. Louis, MO, USA) and human sera from Interstate Blood Bank (Memphis, USA).

### dsRNA-mediated gene knockdown

Individual female *A. gambiae* mosquitoes were injected 1–2 d postemergence as previously described.^10^ Briefly, mosquitoes were injected with 69 nL 3 μg/μL dsRNA solution 3–4 d before receiving a Plasmodium-infected blood meal. dsRNA *A. gambiae* TEP1 were produced using the MEGAscript© RNAi Kit (Ambion, Austin, TX) using DNA templates obtained by PCR using *A. gambiae* cDNA and the primers previously described^10^ with T7 polymerase promoter sites added in the 5'-end. *A. gambiae* TEP1 gene silencing was assessed in whole sugar-fed mosquitoes by quantitative real-time PCR using primers TEP1- qF, TEP1-qR, respectively and were found to be 67% lower by qRT-PCR in dsRNA immune genes injected mosquitoes compared with a dsLacZ-injected control.

### qRT-PCR expression analysis

Total RNA was isolated from 15 *A. gambiae* mosquito midguts 24 h after feeding using TRIzol (Invitrogen) and a modified RNeasy Mini Kit (Qiagen). cDNA was synthesized from 1 μg total RNA using the QuantiTect Reverse Transcription Kit (Qiagen). Gene expression was analyzed by SYBR green qRT-PCR (DyNAmo HS; New England Biolabs) in a CFX96 system using two technical replicates and three biological replicates (qRT-PCR conditions were: 95 °C for 15 min; 44 cycles of 94 °C for 10 s, 50 °C for 20 s, and 60 °C for 30 s; and 60 °C final extension for 5 min). The *P. falciparum* gene Pf10_0203, an ADP ribosylation factor,^10^ was used as an internal reference to normalize each sample.

To determine whether the Pfs47 mD2 antibody prevents the formation of *P. falciparum* ookinetes, we measured the expression of four conserved ookinete-specific genes that have been previously described. These included two secreted adhesive proteins important in mosquito midgut attachment, secreted ookinete adhesive protein (SOAP) and von Willebrand Factor A domain-related protein (WARP), a chitinase (CHT1), and a subtilisin protease-like protein critical for midgut invasion (PIMMS2).^17–20^ The sequences of the primers used are provided below. We calculated fold-change differences in gene expression between *A. gambiae* treated with Pfs47 mD2 antibodies or control IgG antibody using the 2^−ΔΔCt^ method. Gene expression in NF54 gametocytes was measured as a negative control.

qRT-PCR primer sequences used in this study; SOAP_Pf_qPCR_F CAAAGACATCTTCTCGTTCC; SOAP_Pf_qPCR_R TCGGTTTCTTGTTCTGATTT; WARP_Pf_qPCR_F GGTTGGGGAAGAAGGTAAGG; WARP_Pf_qPCR_R CACAAAAATCCCCATCATCA; CHT1_Pf_qPCR_F TCAACCCCTTTTAATCCGAA; CHT1_Pf_qPCR_R ACCCGTCTGCTCTTTTATTT; PIMMS2_Pf_qPCR_F GGGAGAAGGAAAAGGATCTA; PIMMS2_Pf_qPCR_R CTCGGATAATTCTTGTACCG; Pf10_0203_qPCR_F TTGGTGAAGTTGTTACGACT

Pf10_0203_qPCR_R CTCCTACATCCCATACGGTA

### Animals

All animal procedures were performed according to protocols approved by the NIAID and NIH Animal Care and Use Committee. Five to eight weeks old naive, female Balb/c mice were purchased from Charles River (Germantown, MD) and maintained at a facility at the NIH. All procedures were performed in accordance with an animal study protocol approved by the NIAID Animal Care and Use Committee.

### Mice immunization

Groups (*n* = 5) of female BALB/c mice were immunized with a volume of 20 µL containing 5 µg of protein emulsified in magic mouse adjuvant (Creative Diagnostics # CDN-A001) subcutaneously in the ear. The immunized mice were boosted at 2 week intervals three times with the same quantity of protein. Groups of control mice were immunized with adjuvant formulations only. Blood was collected on day 0 (Pre-immune sera) and 2 weeks after each subsequent immunization for analysis of antibodies titer. Hybridoma production was carried out at Antibody and Immunoassay Consultants, Rockville. IgG from mouse serum and hybridoma supernatant was purified as previously described.^21^

### ELISA

Flat-bottom 96-well ELISA plates (Immunolon 4; VWR cat # 62402-959) were coated with 1 µg/ml of recombinant protein diluted in coating buffer (15 mM Na_2_CO_3_, 35 mM NaHCO_3_, pH 9.6) overnight at 4 °C. The plates were washed three times with TBS–0.1% Tween 20 (TBST) and blocked with general block ELISA blocking buffer (ImmunoChemistry cat # 640) for two hours at 37 °C. Polyclonal and monoclonal purified IgG were used at 0.1 µg/ml; animal sera were used at a 1;100 ratio diluted in buffer containing blocking buffer and TBST, added to the antigen-coated wells and incubated for two hours at 37 °C. The plates were then washed three times with TBST and incubated with goat anti-mouse or anti-rabbit immunoglobulin G conjugated to alkaline phosphatase (Seracare cat # 5220-0303) secondary antibodies (0.2 µg/ml) for two hours at 37 °C. The plates were washed again and detection was performed using 100 µL/well of p-nitrophenyl disodium phosphate solution (Sigma 104 phosphatase substrate; 1 tablet per 5 ml of coating buffer). After a 20-min incubation, absorbance was read at 405 nm with VersaMax ELISA plate reader.

### Western Blot

Recombinant proteins were boiled under reducing conditions in SDS-PAGE loading buffer, separated using 12% SDS-PAGE gels and transferred on nitrocellulose membranes. The membrane was blocked for 2 h with 5% non-fat dry milk in TBS–0.1% Tween 20 (TBST). The proteins were detected using 6×-His epitope tag primary antibodies (ThermoFisher cat # MA1-21315) or animal sera at 1:1000 dilutions. The membranes were then washed three times with TBST and incubated with goat anti-mouse or anti-rabbit secondary antibodies conjugated to alkaline phosphatase at 1:5000 dilution. The proteins were then detected using Western Blue Stabilized Substrate for alkaline phosphatase (Promega cat # S3841) after 5 min incubation.

### IFA

Gametocytes, gametes (activated at room temperature for 30 min) were smeared on poly-L-lysine slides and allowed the slides to dry overnight at 4 °C. The slides were then fixed in 4% para-formaldehyde (PFA, in PBS) for 20 min, washed three times (PBS) for 5 min per wash, and blocked and permeabilized in 5% BSA, 0.1% triton (in PBS) prior to antibody staining. We incubated slides overnight at 4 °C with the following primary antibodies: monoclonal mouse anti-Pfs47-mD2 (JH11, IB2, or BM2; 3 mg/mL diluted 1:100), polyclonal rabbit anti-Pfs230 (1 mg/mL diluted 1:1000), polyclonal mouse anti-T-Pfs47 (1.5 mg/mL diluted 1:100), polyclonal mouse anti-Pfs47-mD2 Del1 (1.5 mg/mL diluted 1:100), and polyclonal mouse anti-Pfs47-mD2 Del2 (1.5 mg/mL diluted 1:100). Polyclonal antibodies were preabsorbed with 1% *E. coli* acetone powder. Secondary Alexa Fluor 488 goat anti-mouse (Abcam ab150105) and 594 goat anti-rabbit (Abcam ab150080) antibodies were used at 1:500 dilution and counter-stained with DAPI at 1:1000. Ookinetes were obtained from dissected midguts 24 h post-infection, treated as mentioned above but stained with monoclonal anti-Pfs25 (4B7) (1 mg/mL diluted 1:1000) as primary antibody. Slides as well as ookinete counting were examined using a confocal microscope at 63× magnification.

### Research statement

This research was conducted in accordance with all relevant guidelines and procedures, and has been approved by the National Institutes of Health Intramural Program.

### Data availability

Data that support the findings of this study are available from the corresponding author upon reasonable request.

## Electronic supplementary material

Supplementary Material
