# Supplementary material for: Antibody targeting of a specific region of Pfs47 blocks Plasmodium falciparum malaria transmission
Source: NPJ Vaccines. 2018 Jul 10;3:26. doi: 10.1038/s41541-018-0065-5 (PMC6039440; doi:10.1038/s41541-018-0065-5)
Supplement: Supplementary file 1 — Supplementary Material [file 41541_2018_65_MOESM1_ESM.pdf]

Supplementary Information

Antibody targeting of a specific region of *Pfs47* blocks *Plasmodium falciparum* malaria transmission

Gaspar E. Canepa, Alvaro Molina-Cruz, Lampouguin Yenkoidiok Douti, Eric Calvo, Adeline E. Williams, Marti Burkhardt, Fangni Peng, David Narum, Martin J. Boulanger, Jesus G. Valenzuela, Carolina Barillas-Mury

A

> *P. falciparum* Pfs47 protein sequence (BV-Pfs47)

TQYVCDFYFNPLTNVKPTVVGSSEIYEEVGCTINNPTLGDHIVLICPKKNNGDFSNIEIVPTNCFESHLYSAYKNDSSAYHLEKLDIDKKY
AINSSFSDFYLKILVIPNEYKSHKTIYCRCDNSKTEKNIPGQDKILKGKLGLVKIILRNQYNNIIELEKTKHIIHNKKDTYKYDIKLKESDILMF
YMKEETIVESGNCEEILNTKINLLSNNNVVIKMPSIFINNINCMLSSQDQNNEKNYINLKADKTKHIDGCDFTKPKGKGIYKNGFIINDI
PNEEERICTVHLWNKKNQTIAGIKCPYKLIPPYCFKHVLYEKEIDSQKTYKTFLLSDVLDTPNIEYYGNNKEGMYMLALPTKPEKTNKIR
CICEQGGKKAVMELHIASTSTKYHHHHHH

> *P. falciparum* Pfs47 insect codon optimized DNA sequence (BV-Pfs47)

ACACAATACGTCTGCGACTTCTACTTCAACCCGCTCACTAACGTCAAGCCAACCGTGGTCGGATCTTCGGAAATCTACGAGGAAGTTGGTTGC
ACAATCAACAACCCCACCCTGGGCGACCACATCGTGCTCATCTGTCCTAAGAAGAACAACGGTGACTTCTCTAACATCGAAATCGTGCCCACC
AACTGCTTCGAGTCTCACCTGTACAGCGCTTACAAGAACGACTCCTCTGCCTACCACTTGGAGAAGCTGGACATCGATAAGAAGTACGCTATC
AACAGCTCATTCTCCGACTTCTACCTCAAGATCCTGGTCATCCCCAACGAATACAAGAGCCACAAGACAATCTACTGCCGTTGTGACAACTCAA
AGACCGAGAAGAACATCCCTGGCCAGGATAAGATCCTGAAGGGAAAGCTCGGTTTGGTCAAGATCATCCTCCGCAACCAATACAACAACATC
ATCGAATTGGAGAAGACTAAGCACATCATCCACAACAAGAAGGACACATACAAGTACGATATCAAGTTGAAGGAATCGGACATCCTGATGTT
CTACATGAAGGAGGAAACAATCGTTGAGTCAGGAAACTGCGAGGAAATCCTCAACACTAAGATCAACCTGCTCTCAAACAACAACGTGGTCA
TCAAGATGCCTTCGATCTTCATCAACAACATCAACTGCATGTTGTCGTCCCAGGACCAAAACAACGAGAAGAACTACATCAACCTGAAGGCTG
ATAAGACCAAGCACATCGACGGATGTGATTTCACTAAGCCAAAGGGCAAGGGCATCTACAAGAACGGTTTCATCATCAACGACATCCCGAAC
GAGGAAGAGCGTATCTGCACCGTCCACCTCTGGAACAAGAAGAACCAGACTATCGCCGGCATCAAGTGCCCATACAAGCTGATCCCCCCTTA
CTGTTTCAAGCACGTCCTCTACGAAAAGGAGATCGACTCCCAAAAGACCTACAAGACTTTCTTGCTGTCTGACGTTTTGGATACCCCGAACATC
GAATACTACGGCAACAACAAGGAGGGAATGTACATGCTGGCCCTCCCAACTAAGCCGGAGAAGACAAACAAGATCCGTTGTATCTGCGAGC
AAGGTGGAAAGAAGGCTGTCATGGAACTGCACATCGCTTCTACATCTACAAAATATCATCATCATCATCATCATTGA

B

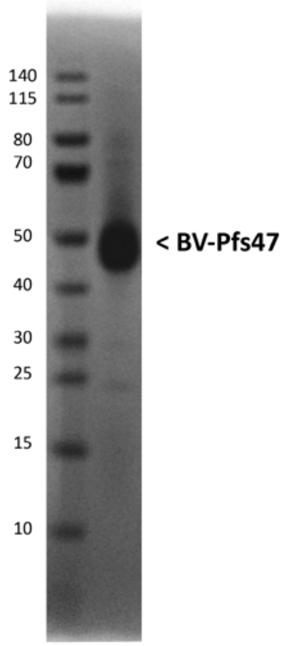

Supplemental figure 1. Expression and Purification of BV-Pfs47 using the baculovirus expression system (**a**) Protein and DNA optimized sequences of BV-Pfs47. His Tag is highlighted in grey. (**b**) Coomassie blue-stained SDS-PAGE gel illustrating purified BV-Pfs47. The unlabeled lane corresponds to the molecular weight standards (KDa).

A

> *P. falciparum* Pfs47 Thioredoxin fusion protein sequence (T-Pfs47)

```
MSDKIIHLTDDSFDTDVLKADGAILVDFWAEWCGPCKMIAPILDEIADEYQGKLTVAKLNIDQNPGTAPKYGIRGIPTLLLFKNGEVAAT
KVGALSKGQLKEFLDANLAGSGSGHSSGLVPRGSGMKETAAAKFERQHMDSPDLGTDDDDKAMADISDPNSSSVDKLAAATQYVCDFYFN
PLTNVKPTVVGSSEIYEEVGCTINNPTLGDHIVLICPKKNNGDFSNIEIVPTNCFESHLYSAYKNDSSAYHLEKLDIDKKYAINSSFSDF
YLKILVIPNEYKSHKTIYCRCDNSKTEKNIPGQDKILKGKLGLVKIILRNQYNNIIELEKTKPIIHNKKDTYKYDIKLKESDILMFYMKE
ETIVESGNCEEILNTKINLLSNNNVVIKMPSIFINNINCMLSSQDQNNEKNYINLKADKTKHIDGCDFTKPKGKGIYKNGFIINDIPNEE
ERICTVHLWNKKNQTIAGIKCPYKLIPPYCFKHVLYEKEIDSQKTYKTFLLSDVLDTPNIEYYGNNKEGMYMLALPTKPEKTNKIRCICE
QGGKKAVMELHIASTSTKYHHHHHH
```

> *P. falciparum* Pfs47 Thioredoxin fusion *E. coli* codon optimized DNA sequence (T-Pfs47)

```
ATGAGCGATAAAATTATTCACCTGACTGACGACAGTTTTGACACGGATGTACTCAAAGCGGACGGGGCGATCCTCGTCGATTTCTGGGCA
GAGTGGTGCGGTCCGTGCAAAATGATCGCCCCGATTCTGGATGAAATCGCTGACGAATATCAGGGCAAACTGACCGTTGCAAAACTGAAC
ATCGATCAAAACCCTGGCACTGCGCCGAAATATGGCATCCGTGGTATCCCGACTCTGCTGCTGTTCAAAAACGGTGAAGTGGCGGCAACC
AAAGTGGGTGCACTGTCTAAAGGTCAGTTGAAAGAGTTCCTCGACGCTAACCTGGCCGGTTCTGGTTCTGGCCATTCTTCTGGTCTGGTG
CCACGCGGTTCTGGTATGAAAGAAACCGCTGCTGCTAAATTCGAACGCCAGCACATGGACAGCCCAGATCTGGGTACCGACGACGACGAC
AAGGCCATGGCTGATATCTCGGATCCGAATTCGAGCTCCGTCGACAAGCTTGCGGCCGCAACACAATACGTATGTGATTTTTATTTTAAT
CCCCTGACTAATGTTAAGCCAACTGTAGTTGGGTCATCTGAAATATACGAAGAAGTTGGATGTACTATAAACAACCCTACGTTGGGTGAC
CATATAGTATTAATATGTCCTAAGAAAAATAATGGAGATTTTAGTAATATAGAAATAGTACCTACTAACTGTTTTGAATCTCATTTATAT
TCTGCTTATAAAAATGATTCCAGCGCATATCATTTAGAAAAATTAGATATCGATAAAAAGTATGCAATAAATTCATCGTTCAGTGATTTC
TATTTAAAAATTTTAGTTATACCTAATGAATATAAAAGTCATAAAACTATATATTGTAGATGTGATAATAGTAAAACGGAAAAAAATATC
CCAGGACAAGATAAAATATTAAAAGGAAAATTAGGATTAGTAAAAATAATTTTAAGAAACCAATATAATAATATAATAGAATTAGAAAAA
ACAAAACCTATTATACATAATAAGAAGGATACATATAAGTATGATATAAAATTAAAAGAAAGTGATATACTTATGTTTTATATGAAAGAA
GAAACTATTGTAGAATCTGGAAATTGTGAAGAAATATTAAATACTAAAATAAATCTATTATCAAATAATAATGTGGTTATAAAAATGCCT
TCCATATTTATAAATAATATTAATTGTATGCTTTCATCTCAAGATCAAAATAATGAAAAAAATTATATAAATCTAAAAGCTGACAAAACA
AAACATATAGATGGGTGTGATTTTACGAAACCTAAAGGTAAAGGTATATACAAAAATGGATTCATAATAAATGATATACCAAATGAAGAA
GAACGTATATGTACTGTTCATCTTTGGAATAAAAAAAATCAAACTATTGCAGGCATTAAATGTCCATATAAATTAATACCACCATATTGT
TTTAAACATGTATTATATGAAAAAGAAATCGATTCGCAAAAGACATATAAAACATTTCTATTAAGTGATGTATTAGATACACCTAATATA
GAATATTATGGAAATAATAAGGAAGGCATGTATATGTTAGCCTTACCAACAAAACCAGAAAAAACAAATAAAATTAGATGTATTTGTGAA
CAAGGTGGAAAAAAAGCAGTAATGGAATTACATATCGCATCTACATCTACAAAATATCACCACCACCACCACCACTGA
```

B

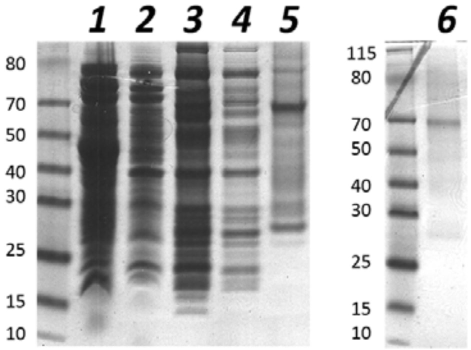

Supplemental figure 2. Expression and Purification of T-Pfs47 expressed in *E. coli* Shuffle T7. (**a**) Protein and DNA optimized sequence of T-Pfs47. Thioredoxin fusion protein from pET32 and His Tag are highlighted in grey. (**b**) Purification of T-Pfs47 (thioredoxin fusion protein); lane 1 - induced cells total lysate; lane 2 - induced cells soluble fraction; lane 3 - first wash of the inclusion bodies (1% triton solution); lane 4 - second wash of the inclusion bodies; lane 5 - inclusion bodies solubilized with 8M urea; lane 6 - purified soluble protein after in-column refolding and nickel affinity purification. The unlabeled lane corresponds to the molecular weight standards (KDa).

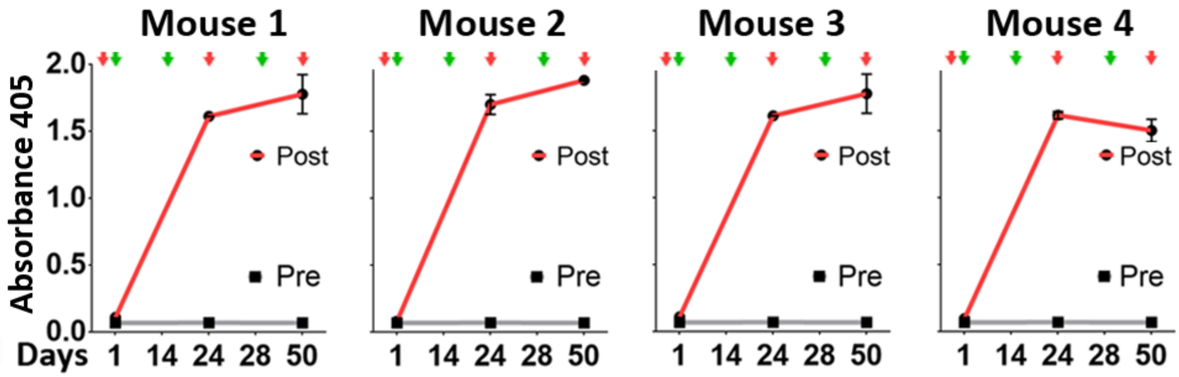

Supplemental figure 3. Immunogenicity of T-Pfs47 in mice. Time course of ELISA titer shows Pfs47 antibody responses after immunization with T-Pfs47 prime and boost in BALB/c mice (Post) compared with control immunization alone (Pre); green arrows indicate vaccination times; red arrows indicate serum sampling.

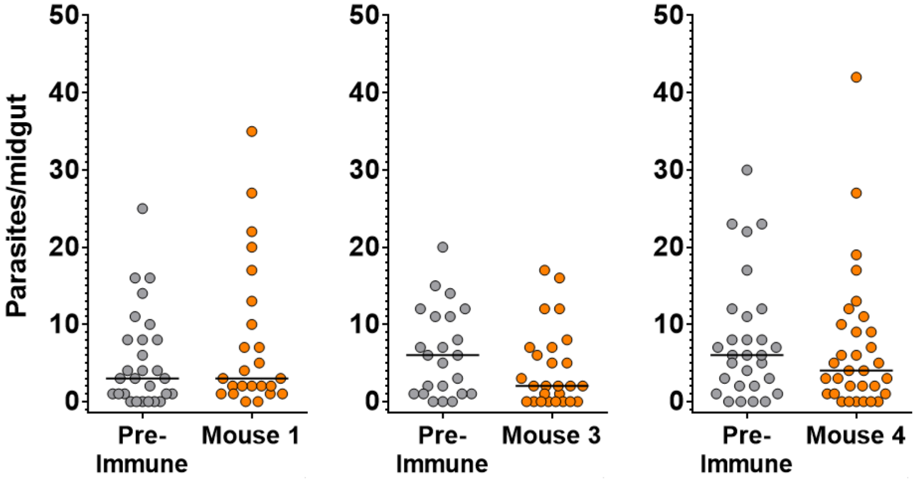

n (midguts)	27	26	23	26	29	32
Prevalence	75	85	87	70	87	82
TBA		0		32		12

Supplemental figure 4. Transmission blocking activity of polyclonal antibodies against T-Pfs47. Submandibular bleeding serum from mouse 1, 3 and mouse 4 at day 50 was used to purify total IgG. 200 µg/ml of IgG was mixed with *P. falciparum* NF54 cultured gametocytes and fed *to A. gambiae* mosquitoes in SMFA. Data points represent the number of oocysts in individual mosquitoes and the lines show the median. The table shows the number of mosquitoes dissected (n), the prevalence and percent inhibition of infection intensity, calculated relative to the mean number of oocysts from control group (Pre-Immune, grey dots). Distribution of parasite numbers in individual mosquitoes between control and experimental groups was compared using the non-parametric Mann Whitney test: no label, no significant difference.

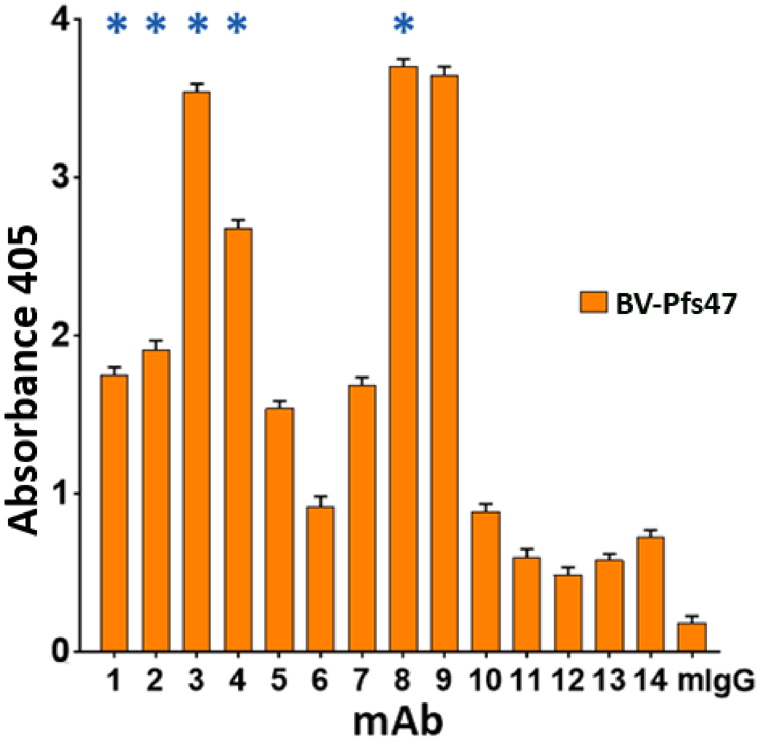

Supplemental figure 5. Characterization of monoclonal antibodies generated against T—Pfs47. Reactivity of monoclonal antibodies 1 to 14 (0.1 µg/ml) generated against T-Pfs47 versus BV-Pfs47 (1 µg/ml) in ELISA. Column and errors bars represent mean OD 405 ± standard deviation of three replicate assays, mIgG corresponds to mouse purified IgG, blue asterisk denotes positive immunofluorescence monoclonal antibodies.

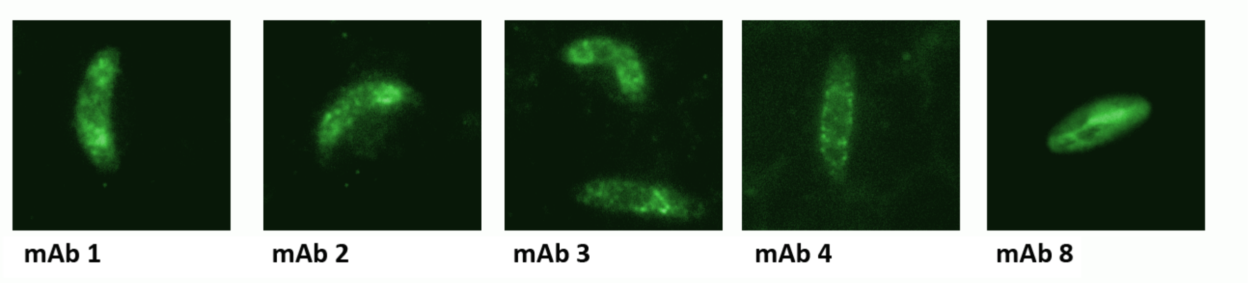

Supplemental figure 6. Immunofluorescence assay of *in vitro* cultured *P. falciparum* NF54 gametocytes stained with purified total IgG of mAbs 1, 2, 3, 4 and 8 generated against T-Pfs47 (green).

A

> *P. falciparum* Pfs47 Domain 1 Thioredoxin fusion protein sequence **(T-Pfs47-D1)**

MSDKIIHLTDDSFDTDVLKADGAILVDFWAEWCGPCKMIAPILDEIADEYQGKLTVAKLNIDQNPGTAPKYGIRGIPTLLLFKNGE
VAATKVGALSKGQLKEFLDANLAGSGSGHSSGLVPRGSGMKETAAAKFERQHMDSPDLGTDDDDKAMADISDPNSSSVDKLAAATQ
YVCDFYFNPLTNVKPTVVGSSEIYEEVGCTINNPTLGDHIVLICPKKNNGDFSNIEIVPTNCFESHLYSAYKNDSSAYHLEKLDID
KKYAINSSFSDFYLKILVIPNEYKSHKTIYCRCDNHHHHHH.

> *P. falciparum* Pfs47 Domain 1 Thioredoxin fusion *E. coli* codon optimized DNA sequence **(T-Pfs47-D1)**

ATGAGCGATAAAATTATTCACCTGACTGACGACAGTTTTGACACGGATGTACTCAAAGCGGACGGGGCGATCCTCGTCGATTTCTG
GGCAGAGTGGTGCGGTCCGTGCAAAATGATCGCCCCGATTCTGGATGAAATCGCTGACGAATATCAGGGCAAACTGACCGTTGCAA
AACTGAACATCGATCAAAACCCTGGCACTGCGCCGAAATATGGCATCCGTGGTATCCCGACTCTGCTGCTGTTCAAAAACGGTGAA
GTGGCGGCAACCAAAGTGGGTGCACTGTCTAAAGGTCAGTTGAAAGAGTTCCTCGACGCTAACCTGGCCGGTTCTGGTTCTGGCCA
TTCTTCTGGTCTGGTGCCACGCGGTTCTGGTATGAAAGAAACCGCTGCTGCTAAATTCGAACGCCAGCACATGGACAGCCCAGATC
TGGGTACCGACGACGACGACAAGGCCATGGCTGATATCTCGGATCCGAATTCGAGCTCCGTCGACAAGCTTGCGGCCGCAACACAA
TACGTATGTGATTTTTATTTTAATCCCCTGACTAATGTTAAGCCAACTGTAGTTGGGTCATCTGAAATATACGAAGAAGTTGGATG
TACTATAAACAACCCTACGTTGGGTGACCATATAGTATTAATATGTCCTAAGAAAAATAATGGAGATTTTAGTAATATAGAAATAG
TACCTACTAACTGTTTTGAATCTCATTTATATTCTGCTTATAAAAATGATTCCAGCGCATATCATTTAGAAAAATTAGATATCGAT
AAAAAGTATGCAATAAATTCATCGTTCAGTGATTTCTATTTAAAAATTTTAGTTATACCTAATGAATATAAAAGTCATAAAACTAT
ATATTGTAGATGTGATAATCACCACCACCACCACCACTGA

B

> *P. falciparum* Pfs47 Domain 3 Thioredoxin fusion protein sequence **(T-Pfs47-D3)**

MSDKIIHLTDDSFDTDVLKADGAILVDFWAEWCGPCKMIAPILDEIADEYQGKLTVAKLNIDQNPGTAPKYGIRGIPTLLLFKNGE
VAATKVGALSKGQLKEFLDANLAGSGSGHSSGLVPRGSGMKETAAAKFERQHMDSPDLGTDDDDKAMADISDPNSSSVDKLAAANN
EKNYINLKADKTKHIDGCDFTKPKGKGIYKNGFIINDIPNEEERICTVHLWNKKNQTIAGIKCPYKLIPPYCFKHVLYEKEIDSQK
TYKTFLLSDVLDTPNIEYYGNNKEGMYMLALPTKPEKTNKIRCICEQGGKKAVMELHIASTSTKYHHHHHH.

> *P. falciparum* Pfs47 Domain 3 Thioredoxin fusion *E. coli* codon optimized DNA sequence **(T-Pfs47-D3)**

ATGAGCGATAAAATTATTCACCTGACTGACGACAGTTTTGACACGGATGTACTCAAAGCGGACGGGGCGATCCTCGTCGATTTCTG
GGCAGAGTGGTGCGGTCCGTGCAAAATGATCGCCCCGATTCTGGATGAAATCGCTGACGAATATCAGGGCAAACTGACCGTTGCAA
AACTGAACATCGATCAAAACCCTGGCACTGCGCCGAAATATGGCATCCGTGGTATCCCGACTCTGCTGCTGTTCAAAAACGGTGAA
GTGGCGGCAACCAAAGTGGGTGCACTGTCTAAAGGTCAGTTGAAAGAGTTCCTCGACGCTAACCTGGCCGGTTCTGGTTCTGGCCA
TTCTTCTGGTCTGGTGCCACGCGGTTCTGGTATGAAAGAAACCGCTGCTGCTAAATTCGAACGCCAGCACATGGACAGCCCAGATC
TGGGTACCGACGACGACGACAAGGCCATGGCTGATATCTCGGATCCGAATTCGAGCTCCGTCGACAAGCTTGCGGCCGCAAATAAT
GAAAAAAATTATATAAATCTAAAAGCTGACAAAACAAAACATATAGATGGGTGTGATTTTACGAAACCTAAAGGTAAAGGTATATA
CAAAAATGGATTCATAATAAATGATATACCAAATGAAGAAGAACGTATATGTACTGTTCATCTTTGGAATAAAAAAAATCAAACTA
TTGCAGGCATTAAATGTCCATATAAATTAATACCACCATATTGTTTTAAACATGTATTATATGAAAAAGAAATCGATTCGCAAAAG
ACATATAAAACATTTCTATTAAGTGATGTATTAGATACACCTAATATAGAATATTATGGAAATAATAAGGAAGGCATGTATATGTT
AGCCTTACCAACAAAACCAGAAAAAACAAATAAAATTAGATGTATTTGTGAACAAGGTGGAAAAAAAGCAGTAATGGAATTACATA
TCGCATCTACATCTACAAAATATCACCACCACCACCACCACTGA

C

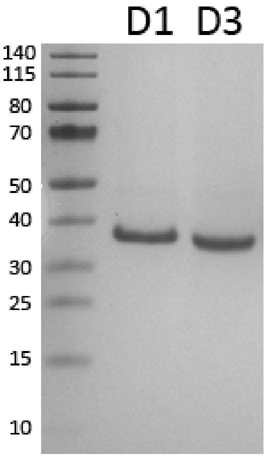

Supplemental figure 7. Expression and purification of T-Pfs47-D1 and T-Pfs47-D3). (Protein and DNA optimized sequences of (**a**) T-Pfs47-D1 and (**b**) Thioredoxin fusion protein from pET32 and His Tag are highlighted in grey. (**c**) Coomassie blue-stained SDS-PAGE gel illustrating the purified T-Pfs47-D1 and T-Pfs47-D3. The unlabeled lane corresponds to the molecular weight standards (KDa).

A

> *P. falciparum* Pfs47 D1-D3 construct (Thr32-Asn154 GSGGSG Asn268-Ala414) protein sequence **(BV-Pfs47-D1-D3)**

TQYVCDFYFNPLTNVKPTVVGSSEIYEEVGCTINNPTLGDHIVLICPKKNNGDFSNIEIVPTNCFESHLYSAYKNDSSAYHLEKLDIDKKYAINSSFSDFYL
KILVIPNEYKSHKTIYCRCDNGSGGSGNNEKNYINLKADKTKHIDGCDFTKPKGKGIYKNGFIINDIPNEEERICTVHLWNKKNQTIAGIKCPYKLIPPYC
FKHVLYEKEIDSQKTYKTFLLSDVLDTPNIEYYGNNKEGMYMLALPTKPEKTNKIRCICEQGGKKAVMELHIASTSTKYHHHHHH

> *P. falciparum* Pfs47 D1-D3 construct (Thr32-Asn154 GSGGSG Asn268-Ala414) insect codon optimized DNA sequence **(BV-Pfs47-D1-D3)**

AACAACGAGAAGAACTACATCAACCTGAAGGCTGATAAGACCAAGCACATCGACGGATGTGATTTCACTAAGCCAAAGGGCAAGGGCATCT
ACAAGAACGGTTTCATCATCAACGACATCCCGAACGAGGAAGAGCGTATCTGCACCGTCCACCTCTGGAACAAGAAGAACCAGACTATCGCC
GGCATCAAGTGCCCATACAAGCTGATCCCCCCTTACTGTTTCAAGCACGTCCTCTACGAAAAGGAGATCGACTCCCAAAAGACCTACAAGACT
TTCTTGCTGTCTGACGTTTTGGATACCCCGAACATCGAATACTACGGCAACAACAAGGAGGGAATGTACATGCTGGCCCTCCCAACTAAGCCG
GAGAAGACAAACAAGATCCGTTGTATCTGCGAGCAAGGTGGAAAGAAGGCTGTCATGGAACTGCACATCGCTTCTACATCTACAAAATATCA
TCATCATCATCATCATTGA

B

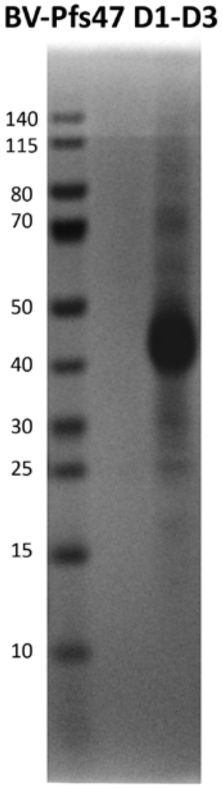

Supplemental figure 8. Expression and purification BV-Pfs47 D1-D3. (**a**) Protein and DNA optimized sequences of BV-Pfs47-D1-D3. His Tag is highlighted in grey. (**b**) Coomassie blue-stained SDS-PAGE gel illustrating the purified BV-Pfs47-D1-D3. The unlabeled lane corresponds to the molecular weight standards (KDa).

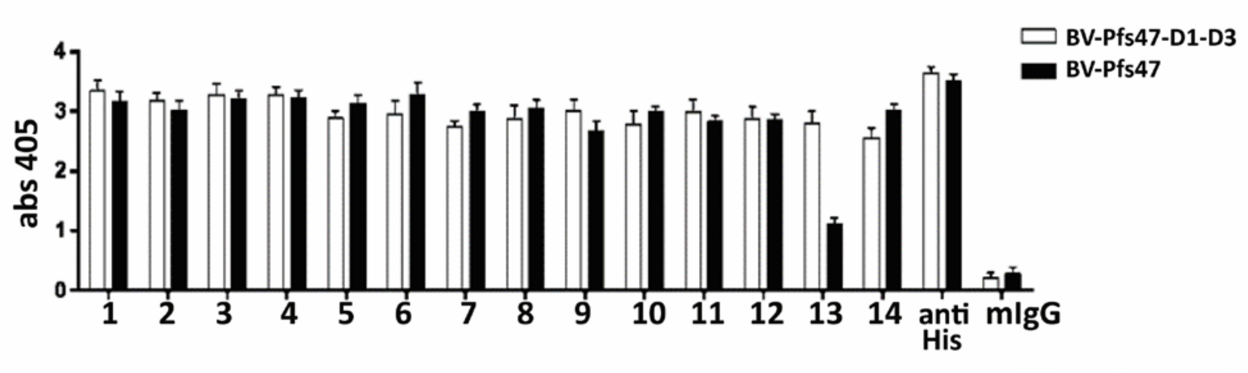

Supplemental figure 9. Antigen mapping of monoclonal antibodies generated against T-Pfs47. Reactivity of monoclonal antibodies 1 to 14 (0.1 µg/ml) generated against T-Pfs47 detecting recombinant BV-Pfs47 (1 µg/ml) (black bars) or BV-Pfs47-D1-D3 (1 µg/ml) (white bars) in ELISA. Column and errors bars represent mean OD 405 ± standard deviation of three replicate assays. mIgG, normal mouse purified IgG.

A

P. falciparum Pfs47 modified Domain 2 protein sequence **(Pfs47-mD2)**

MSKTEKNIPGQDKILKGKLGLVKIILRNQYNNIIELEKTKHIIHNKKDTYKYDIKLKESDILMFYMKEETIVESGNAEEILNTKINLLSNNNVVIKMPSIFIN
NINAMLSSQHHHHHH.

P. falciparum Pfs47 modified Domain 2 *E. coli* codon optimized DNA sequence **(Pfs47-mD2)**

ATGAGCAAAACCGAGAAAAACATTCCGGGTCAAGATAAAATCCTGAAAGGCAAACTGGGTCTGGTGAAAATCATCCTGCGTAATCAATACAA
CAACATCATTGAGCTGGAAAAGACCAAACACATCATTCACAACAAGAAAGACACCTACAAGTATGATATCAAGCTGAAAGAGAGCGACATTC
TGATGTTCTATATGAAAGAGGAAACCATCGTGGAAAGCGGTAACGCGGAGGAAATCCTGAACACCAAGATTAACCTGCTGAGCAACAACAA
CGTGGTTATTAAAATGCCGAGCATTTTTATCAACAATATCAACGCGATGCTGAGCAGCCAACACCACCACCACCACCACTAA

B

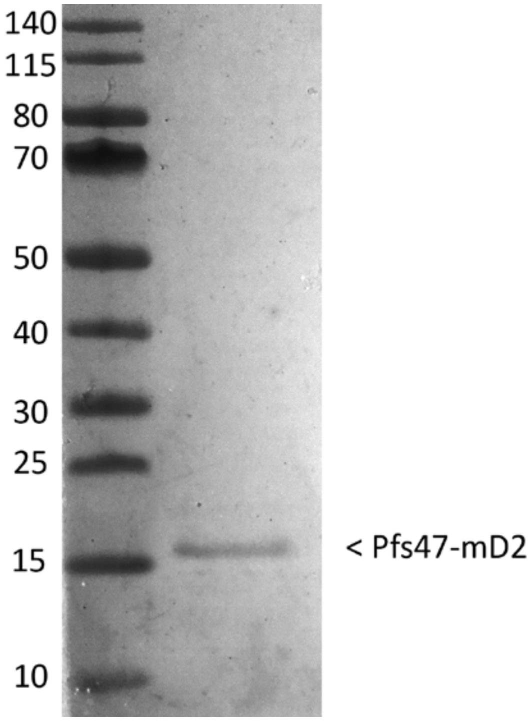

Supplemental figure 10. Expression of modified Pfs47 D2 (Pfs47-mD2) in *E. coli*. (**a**) Protein and DNA optimized sequences of Pfs47-mD2 showing cysteine replacements to alanines in red (C230A, C260A). Six-histidine tag are highlighted in grey. (**b**) Coomassie blue-stained SDS-PAGE gel illustrating the purified Pfs47-mD2. The unlabeled lanes show molecular weight standards (KDa).

A

**Pfs47-mD2**	**MSKTEKNIPGQDKILKGKLGLVKIILRNQYNNIIELEKTKHIIHNKKDTYKYDIKLKESDILMFYMKEETIVESGNAEEILNTKINLLSNNNVVIKMPSIFINNINAMLSSQHHHHHH**
**Del1**	**MILRNQYNNIIELEKTKHIIHNKKDTYKYDIKLKESDILMFYMKEETIVESGNAEEILNTKINLLSNNNVVIKMPSIFINNINAMLSSQHHHHHH**
**Del2**	**MILRNQYNNIIELEKTKHIIHNKKDTYKYDIKLKESDILMFYMKEETIVESGNHHHHHH**
**Del3**	**MSKTEKNIPGQDKILKGKLGLVKIILRNHHHHHH**

B

P. falciparum Pfs47 modified Domain 2 Deletion 1 protein sequence **(Del1)**
MILRNQYNNIIELEKTKHIIHNKKDTYKYDIKLKESDILMFYMKEETIVESGNAEEILNTKINLLSNNNVVIKMPSIFINNINAMLSSQHHHHHH.

P. falciparum Pfs47 modified Domain 2 Deletion 1 *E. coli* codon optimized DNA sequence **(Del1)**
ATGATCCTCCGCAACCAATACAACAACATCATCGAATTGGAGAAGACTAAGCACATCATCCACAACAAGAAGGACACATACAAGTACGATATCAAGTTGAAGGAATCGGACATCCTGATGTTCTACATGAAGGAGGAAACAATCGTTGAGTCAGGAAACGCCGAGGAAATCCTCAACACTAAGATCAACCTGCTCTCAAACAACAACGTGGTCATCAAGATGCCTTCGATCTTCATCAACAACATCAACGCCATGTTGTCGTCCCAGCACCACCACCACCACCACTAA

P. falciparum Pfs47 modified Domain 2 Deletion 2 protein sequence **(Del2)**
MILRNQYNNIIELEKTKHIIHNKKDTYKYDIKLKESDILMFYMKEETIVESGNHHHHHH.

P. falciparum Pfs47 modified Domain 2 Deletion 2 *E. coli* codon optimized DNA sequence (Del2)
ATGATCCTCCGCAACCAATACAACAACATCATCGAATTGGAGAAGACTAAGCACATCATCCACAACAAGAAGGACACATACAAGTACGATATCAAGTTGAAGGAATCGGACATCCTGATGTTCTACATGAAGGAGGAAACAATCGTTGAGTCAGGAAACCACCACCACCACCACCACTAA

P. falciparum Pfs47 modified Domain 2 Deletion 3 protein sequence **(Del3)**
MSKTEKNIPGQDKILKGKLGLVKIILRNHHHHHH.

P. falciparum Pfs47 modified Domain 2 Deletion 3 *E. coli* codon optimized DNA sequence **(Del3)**
ATGAGCAAAACCGAGAAAAACATTCCGGGTCAAGATAAAATCCTGAAAGGCAAACTGGGTCTGGTGAAAATCATCCTGCGTAATCACCACCACCACCACCACTAA

C

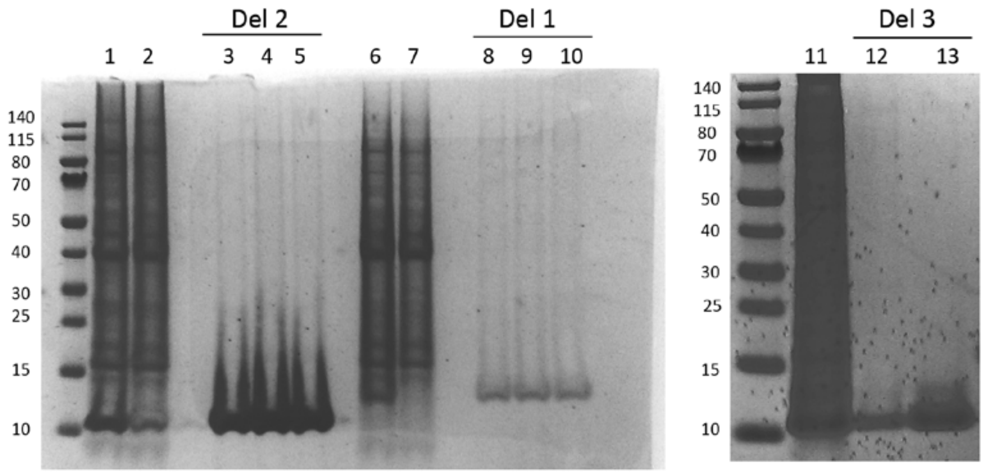

Supplemental figure 11. (**a**) Schematic representation of Pfs47-mD2 and the deletions generated (Del1, Del2, and Del3). (**b**) Protein and DNA optimized sequences of Del1, Del2, and Del3 showing cysteine replacements to alanines in red (C230A, C260A). Six-histidine tag are highlighted in grey. (**c**) Coomassie blue-stained SDS-

PAGE gel illustrating the purified Del1, Del2, and Del3. The gel shows the input sample before nickel affinity purification (lane 1, Del2; lane 6, Del1; lane 11, Del3), the flow through after nickel purification (lane 2, Del2; lane 7, Del1) and serial elution from the Nickel affinity purification column (lanes 3, 4 and 5, Del2; lanes 8, 9 and 10, Del1; lanes 12 and 13, Del3). The unlabeled lanes show molecular weight standards.

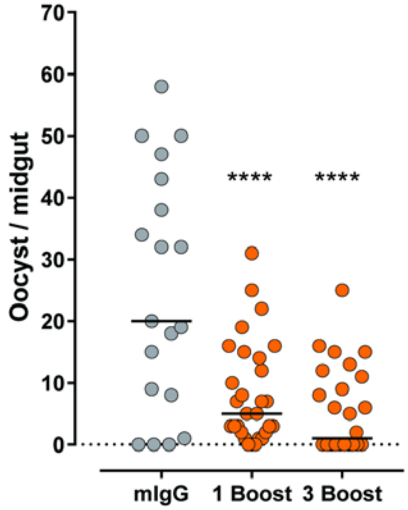

n (midguts)	19	29	26
Prevelance	85	90	50
TBA		67	78

Supplemental figure 12. Transmission blocking activity of polyclonal sera against Pfs47-mD2 Del2 (200ug/ml) after 1 or 3 boosts. Data points represent the number of oocysts in individual mosquitoes and the lines show the median. The table shows the number of mosquitoes dissected (n), the prevalence and percent inhibition of infection intensity, calculated relative to the mean number of oocysts from control group (mIgG, grey dots). Medians were compared using the Mann–Whitney test: no label, no significant difference; ****P < 0.0001.

```
Hp1   ILRNQYNNIIELEKTKHIIHNKKDTYKYDIKLKESDILMFYMKEETIVESGN
Hp2   ILRNQYNNIIELEKTKPIIHNKKDTYKYDIKLKESDILMFYMKEETIVESGN
Hp3   ILRNQYNNIIELEKTKHIIHNKKDTYKYDIKLKESDILMFYIKEETIVESGN
Hp4   ILRNQYNNIIDLEKTKHIIHNKKDTYKYDIKLKESDILMFYMKEETIVESGN
Hp5   VLRNQYNNIIELEKTKHIIHNKKDTYKYDIKLKESDILMFYMKEETIVESGN
Hp6   ILRNQYNNIIELEKTKHIIHNKKDTYKYDIKLKESDILMFYMKEETNVESGN
Hp7   ILRNQYNNKIELEKTKHIIHNKKDTYKYDIKLKESDILMFYMKEETNVESGN
```

Supplemental figure 13. Sequence diversity of Pfs47-D2 Del 2 in natural populations of *P. falciparum*. Analysis of the Pfs47 Domain 2-Del2 region from 364 *P. falciparum* isolates from humans collected around the world indicate that there are only seven haplotypes (Hp 1-7) for this 52 aa vaccine target region. Six of them differ by a single amino acid (98% identity), and one by two amino acids (96% identity). Linear B cell epitopes predicted by the Bepipred program are indicated in grey. Amino acid differences are indicated in darl grey and bold.

Construct	Fusion	System	Detected Expression (WB, ELISA)
pVR2010-Pfs47	No	Human cells, HEK293i	NO
	Heptamerization Domain	Human cells, HEK293i	NO
pBIO-Pfs47 (AVEXIS)	Cd4 (pentamerization)	Human cells, HEK293i	NO
pLAC-Pfs47 (AVEXIS)	Cd4 (pentamerization) beta-lactamase	Human cells, HEK293i	NO
pRLNULL-Pfs47	No	Insect cells, Sua5.1	NO
		Insect cells, Aag2	NO
BV-Pfs47 Baculovirus	No	Insect cells, SF9	YES<br>Low yield<br>(~100 µg/L)
pET32-T-Pfs47	Thioredoxin	E. coli, Shuffle T7	YES<br>Yield ~1 mg/L

Supplemental table 1. Summary of the plasmid constructs used for optimization of the expression of *Plasmodium falciparum* Pfs47. In all the strategies, the nucleotide sequence of Pfs47 coding region was codon-optimized to suit the expression system used. The predicted signal peptide and GPI anchor were removed, and a histidine tag was added at the C-terminus for affinity purification. Expression of several different Pfs47-encoding constructs in mammalian cell lines (pVR2010, pBIO and pLAC-based plasmids in Human Embryonic Kidney cells) or insect cell lines (Sua5.1 from *Anopheles gambiae* and Aag2 from *Aedes aegipty* cells) were all unsuccessful. The expression in Sf9 insect cells using a Baculovirus expression system resulted in very low yield of the recombinant protein (about 100 µg/L of harvested supernatant). High yields of expression were obtained only when using the pET32 system in *Escherichia coli*, in-column refolding of inclusion bodies followed by affinity purification. WB, western-blot.

Experiment 1	mIgG	4B7 (anti Pfs25)	mAb 1	mAb 2	mAb 4	mAb 5	mAb 8
n (midguts)	27	30	30	35	23	21	30
Oocyst/mgt Median	25	2**	14.5	15	33	8*	35.5
Prevalence (%)	93	77	80	89	96	86	90
% Inhibition (mean)		57	13	21	0	28	0

mAb

Experiment 2	mIgG	4B7 (anti Pfs25)	mAb 1	mAb 2	mAb 4	mAb 6	mAb 7	mAb 8
n (midguts)	42	38	36	32	42	21	30	38
Oocyst/mgt Median	1	0	0	25	0	1	1	1
Prevalence (%)	57	16	39	0	36	62	57	58
% Inhibition (mean)		86	0	54	0	0	0	0

mAb

Experiment 3	mIgG	4B7 (anti Pfs25)	mAb 1	mAb 2	mAb 3	mAb 4	mAb 5
n (midguts)	29	39	34	30	33	26	21
Oocyst/mgt Median	3	0****	0**	2**	1	1***	0**
Prevalence (%)	90	10	44	57	70	46	57
% Inhibition (mean)		98	45	40	19	68	25

mAb

Experiment 4	mIgG	4B7 (anti Pfs25)	mAb 3	mAb 4	mAb 5	mAb 9	mAb 10	mAb 13	mAb 14
n (midguts)	30	37	31	38	44	51	28	30	30
Oocyst/mgt Median	4	0	2	4	3	4	3.5	2.5	2.5
Prevalence (%)	87	17	75	85	87	87	83	70	70
% Inhibition (mean)		96	0	20	12	0	5	9	9

mAb

Experiment 5	mIgG	4B7 (anti Pfs25)	mAb 3	mAb 4	mAb 8	mAb 9	mAb 10	mAb 11	mAb 12
n (midguts)	26	30	30	24	38	26	30	26	30
Oocyst/mgt Median	48	2	28	9***	20	56	17	16	33
Prevalence (%)	81	24	67	50	56	77	57	58	64
% Inhibition (mean)		87	10	65	21	5	38	10	0

mAb

Experiment 6	mIgG	4B7 (anti Pfs25)	mAb 4	mAb 5	mAb 9	mAb 10	mAb 11	mAb 12	mAb 13
n (midguts)	33	30	27	21	37	38	28	32	33
Oocyst/mgt Median	35	2	14	16	12	49.5	20	32.5	18
Prevalence (%)	88	24	63	53	55	77	65	75	61
% Inhibition (mean)		87	33	14	38	0	7	0	26

Supplemental table 2. Transmission blocking activity of monoclonal antibodies against T-Pfs47. Purified IgG (200 µg/ml) from mAbs obtained after immunization with T-Pfs47 was mixed with *P. falciparum* NF54 cultured gametocytes and fed to *A. gambiae* mosquitoes in SMFA. The table represent at least six independent SMFA using mAbs 1-14. The table shows the number of mosquitoes dissected (n), the Oocyst/midgut median, the prevalence and percent inhibition of infection intensity, calculated relative to the mean number of oocysts from control group (mIgG,

grey boxes). 4B7 (yellow boxes) correspond to a mAbs against Pfs25 used as control. Orange boxes corresponds to significative TBA. Distribution of parasite numbers in individual mosquitoes between control and experimental groups was compared using the non-parametric Mann Whitney test: no label, no significant difference; *P < 0.05; **P < 0.01; ***P < 0.001; ****P < 0.0001.

	Experiment 1		*Experiment 2*		*Experiment 3*		*Experiment 4*	
	mIgG	pool	mIgG	pool	mIgG	pool	mIgG	pool
n (midguts)	40	27	28	38	27	29	27	33
Oocyst/mgt Median	3	1*	6	1**	5	3	5	4
Prevalence (%)	68	56	90	64	86	83	85	84
% Inhibition (mean)		71		60		35		40

Supplemental table 3. Transmission blocking activity of pooled monoclonal antibodies against T-Pfs47. Pooled purified IgG (200 µg/ml) from mAbs 2, 3, 4, and 8 was mixed with *P. falciparum NF54* cultured gametocytes and fed to *A. gambiae* mosquitoes in SMFA. The table represent at least four independent SMFA. The table shows the number of mosquitoes dissected (n), the Oocyst/midgut median, the prevalence and percent inhibition of infection intensity, calculated relative to the mean number of oocysts from control group (mIgG, grey boxes). Orange boxes corresponds to significative TBA. Distribution of parasite numbers in individual mosquitoes between control and experimental groups was compared using the non-parametric Mann Whitney test: no label, no significant difference; *$P < 0.05$; **$P < 0.01$.

	Plasmodium falciparum NF54 + Anopheles gambiae G3					
mAb anti Pfs47 mD2	Experiment 1	Experiment 2	Experiment 3	Experiment 4		
**mIgG**						
n (midguts)	35	26	33	24		
Oocyst/mgt Median	60	25	2	1		**ELISA-WB**
Prevalence (%)	91	81	73	77		
**IB2**					**IB2**	
n (midguts)	33	22	43	26	+	Elisa Pfs47 mD2
Oocyst/mgt Median	14****	2****	0**	0***	-	Elisa BV-Pfs47
Prevalence (%)	79	46	35	24	+	WB Pfs47 mD2
% Inhibition (mean)	70	88	66	84	+	WB BV-Pfs47
**BM2**					**BM2**	
n (midguts)			31	26	+	Elisa Pfs47 mD2
Oocyst/mgt Median			0****	0**	+	Elisa BV-Pfs47
Prevalence (%)			10	20	-	WB Pfs47 mD2
% Inhibition (mean)			94	74	+	WB BV-Pfs47
**AB1**					**AB1**	
n (midguts)	33	29			+	Elisa Pfs47 mD2
Oocyst/mgt Median	29*	2****			-	Elisa BV-Pfs47
Prevalence (%)	97	38			+	WB Pfs47 mD2
% Inhibition (mean)	37	88			-	WB BV-Pfs47
**EB1**					**EB1**	
n (midguts)	30	32	35		+	Elisa Pfs47 mD2
Oocyst/mgt Median	13****	10.5	2		-	Elisa BV-Pfs47
Prevalence (%)	67	82	73		+	WB Pfs47 mD2
% Inhibition (mean)	67	28	0		-	WB BV-Pfs47
**HG3**					**HG3**	
n (midguts)	25	30			+	Elisa Pfs47 mD2
Oocyst/mgt Median	16****	4***			-	Elisa BV-Pfs47
Prevalence (%)	76	70			+	WB Pfs47 mD2
% Inhibition (mean)	68	78			+	WB BV-Pfs47
**BF1**					**BF1**	
n (midguts)	36	33			+	Elisa Pfs47 mD2
Oocyst/mgt Median	23***	0****			+	Elisa BV-Pfs47
Prevalence (%)	77	31			+	WB Pfs47 mD2
% Inhibition (mean)	51	86			+	WB BV-Pfs47
**JH11**					**JH11**	
n (midguts)				28	+	Elisa Pfs47 mD2
Oocyst/mgt Median				4.5*	+	Elisa BV-Pfs47
Prevalence (%)				75	+	WB Pfs47 mD2
% Inhibition (mean)				-240	+	WB BV-Pfs47
**EH3**					**EH3**	
n (midguts)				36	+	Elisa Pfs47 mD2
Oocyst/mgt Median				0	+	Elisa BV-Pfs47
Prevalence (%)				48	-	WB Pfs47 mD2
% Inhibition (mean)				-22	+	WB BV-Pfs47

Supplemental table 4. Transmission blocking activity and characterization by ELISA and Western Blot of monoclonal antibodies generated against Pfs47-mD2. Purified IgG (200 µg/ml) from mAbs obtained after immunization with Pfs47-mD2 was mixed with *P. falciparum NF54* cultured

gametocytes and fed to *A. gambiae* mosquitoes in SMFA. The table represents at least four independent SMFA using mAbs IB2, AB1, HG3, BF1, BM2, EB1, JH11 and EH3. The table shows the number of mosquitoes dissected (n), the Oocyst/midgut median, the prevalence and percent inhibition of infection intensity, calculated relative to the mean number of oocysts from control group (mIgG). Distribution of parasite numbers in individual mosquitoes between control and experimental groups was compared using the non-parametric Mann Whitney test: no label, no significant difference; *P < 0.05; **P < 0.01; ***P < 0.001; ****P < 0.0001. The right column represents a summary of the reactivity of these mAbs against the antigens BV-Pfs47 and Pfs47-mD2 on western Blots and ELISA assays.

Image Processing.

All gel images derive from the same experiment and were processed in parallel.
